# Genotyping strategies for single-step genomic predictions in a simulated sheep population under different scenarios of pedigree error types

**DOI:** 10.3389/fgene.2025.1697103

**Published:** 2025-11-10

**Authors:** Artur O. Rocha, Leonardo S. Gloria, Andre C. Araujo, Hui Wen, Carrie S. Wilson, Bradley A. Freking, Thomas W. Murphy, Joan M. Burke, Ronald M. Lewis, Luiz F. Brito

**Affiliations:** 1 Department of Animal Sciences, Purdue University, West Lafayette, IN, United States; 2 The Pig Improvement Company, Genus PIC, Hendersonville, TN, United States; 3 AcuFast Swine, AcuFast™, Saskatoon, SK, Canada; 4 USDA, ARS, Range Sheep Production Efficiency Research Unit, Dubois, ID, United States; 5 USDA, ARS, Roman L. Hruska U.S. Meat Animal Research Center, Clay Center, NE, United States; 6 USDA, ARS, Dale Bumpers Small Farms Research Center, Booneville, AR, United States; 7 Department of Animal Science, University of Nebraska-Lincoln, Lincoln, NE, United States

**Keywords:** composite breed, pedigree errors, Ovine, genotyping strategy, genomic prediction, ssGBLUP

## Abstract

Genomic predictions provide more accurate estimated breeding values (EBV) in younger animals. However, sheep reference populations are still small and if the animals included in the reference populations are not chosen carefully, genomic predictions may be biased. In this context, we compared genotyping strategies varying in the proportion of animals genotyped (using a 50K SNP panel) and the extent of pedigree errors (misidentified sires or missing information) on accuracy, bias, and dispersion of genomically-enhanced EBV (GEBV). We simulated a composite sheep population mimicking the formation and flock structure of the Katahdin breed using the AlphaSimR package. Sixteen flocks with an effective population size of 103 were simulated for two traits with heritabilities of 0.35 and 0.10. Breeding values were predicted with Best Linear Unbiased Prediction (BLUP) and Single-step Genomic BLUP (ssGBLUP). Scenarios included combinations of 0%–100% males or females genotyped, 0%–20% pedigree errors, and three genotyping strategies (random, highest EBV, or highest phenotypic values). The final population (18,717 animals) was divided into training and validation sets for calculating validation statistics of GEBV. Genomic prediction accuracy significantly improved with random genotyping, outperforming phenotype and EBV-based strategies by up to 19%. Pedigree errors reduced GEBV accuracy while increasing bias and dispersion. Missing pedigree information impacted results more than misidentified sires. Increasing the proportion of animals genotyped improved GEBV prediction metrics, with random genotyping yielding higher accuracies, lower biases, and dispersion closer to 1 (desirable). Prioritizing the genotyping of males up to 10% of the population before incorporating females enhanced the accuracy of GEBV. Genomic information mitigated some pedigree error effects. However, selective genotyping increased GEBV bias and dispersion, and reduced prediction accuracy. Compared to random genotyping, selective genotyping captured less genomic diversity, limiting the effectiveness of the reference population. Similar conclusions were obtained for both trait heritability levels. These findings highlight the importance of genotyping strategies when implementing genomic selection in sheep and the usefulness of genomic information for minimizing the impact of pedigree errors.

## 1 Introduction

Sheep production plays a vital role in ensuring a reliable and diversified supply of food and fiber in North America. In more recent decades, there has been a growing emphasis toward meat production, with structured breeding programs contributing to improvements in productivity. For instance, the National Sheep Improvement Program (NSIP), established in the late 1980s, has provided across-flock genetic evaluations for growth, reproduction, and carcass traits using the Best Linear Unbiased Predictions (BLUP) methodology. These efforts have demonstrated measurable genetic gains and updated genetic parameters, such as those reported in Polypay, Suffolk and Katahdin sheep for growth, reproduction, wool, carcass, and longevity traits (Notter, 1998; Pinto et al., 2025a; Saleem et al., 2025). Such achievements underscore the effectiveness of pedigree-based selection and provide a strong foundation for further advances through genomic selection.

Genomic selection (Meuwissen et al., 2001) is now commonly used in sheep breeding programs around the world, including Australia and New Zealand (Daetwyler et al., 2010; Woolley et al., 2023), France (Baloche et al., 2014), United Kingdom (Kaseja et al., 2023), and, more recently, in other countries such as the U.S. (McMillan et al., 2022; Rocha et al., 2023). Genomic selection enables more accurate prediction of breeding values than pedigree-based methods. This improvement is particularly evident in species with larger reference populations (Meuwissen et al., 2001; Brito et al., 2017; Guarini et al., 2018). However, in small ruminants such as sheep, the effectiveness of genomic selection on realized genetic gain depends strongly on factors such as the size of the reference population and the intensity of selection, and may not surpass traditional selection strategies when these are limited (Ibañez-Escriche and Gonzalez-Recio, 2011; Brito et al., 2017). When incorporating genomic information, the pedigree relationship matrix used to obtain estimated breeding values (EBV) from BLUP is replaced or, more often, combined with the genomic relationship matrix to predict genomically-enhanced EBV (GEBV) with the Single-step Genomic BLUP (ssGBLUP) method (Legarra et al., 2009; Aguilar et al., 2010; Christensen and Lund, 2010). However, before implementing genomic selection, validation studies are generally conducted, to assess the quality of the genomic predictions (Legarra and Reverter, 2018).

The incremental increase in the accuracy of GEBV is particularly important for traits that are measured late in life, sex-limited, traits with low heritability, and for animals lacking their own phenotypic records (Meuwissen et al., 2001; Daetwyler et al., 2012; Brown et al., 2018; Brito et al., 2020). The performance of genomic predictions is highly dependent on the size and composition of the reference population, including the number of genotyped animals, number of phenotypes available for genotyped animals, the genetic relationship of reference individuals with the selection candidates, and how genetically representative these reference animals are of the target population (Lund et al., 2016; van den Berg et al., 2019). Genomic prediction offers clear benefits by improving accuracy, particularly in sheep populations where pedigree-based EBV often have low initial accuracy. For example, in U.S. Rambouillet sheep, 41% and 62% improvement in GEBV accuracies were reported for postweaning body weight and yearling body weight when compared to EBV (Araujo et al., 2023). For composite sheep populations, the gains in GEBV accuracy might be lower due to their higher effective population size and lower connectedness structure compared to other sheep breeds or other species (Rupp et al., 2016; Araujo et al., 2021; Nilson et al., 2024). Furthermore, the cost of genotyping, small flock sizes, limited use of artificial insemination, and relatively higher proportions of misidentified animals or missing information in the pedigree records compared to other livestock species make the implementation of genomic selection in sheep more challenging (van der Werf et al., 2014; Nel et al., 2023).

Up to a certain level, the larger the reference population, the higher the GEBV prediction accuracy. However, due to limited economic resources for genotyping in most sheep breeding programs, it is not feasible to genotype all or even most selection candidates. Depending on the genotyping strategy used, the prediction bias might increase and the GEBV accuracy might not improve as expected (Schöpke and Swalve, 2016). Several studies based on real and simulated data have evaluated strategies to design reference populations to find an optimum scenario that maximizes the GEBV prediction accuracy while minimizing the source of biases, but none have been conducted in U.S. sheep (van der Werf et al., 2014; Cesarani et al., 2019; Granado-Tajada et al., 2021; Liu et al., 2023). U.S. flocks are typically smaller, more dispersed across diverse production environments, and involve a wider range of breeds and composite populations compared to many intensively selected or nucleus-based breeding schemes found elsewhere.

Unknown parentage and misidentified animals in the pedigree are challenges in any genetic improvement program, particularly affecting pedigree-based BLUP, where errors may remain uncorrected. These issues can lead to lower genetic progress and reduced EBV theoretical accuracy (Nwogwugwu et al., 2020). While genomic information can mitigate some of these effects, pedigree errors may still impact the effectiveness of genomic prediction models. In U.S. dairy cattle populations, up to 14% of genotyped females had misidentified sires in 2012 (Wiggans et al., 2012). Up to 6% of pedigree errors were reported for UK Texel sheep (Kaseja et al., 2022). These errors can cause a mismatch between the pedigree (**A**) and genomic (**G**) relationship matrices, leading to a reduction in the correlation between their off-diagonal elements. This weakened alignment between pedigree-based and genomic relationships can result in more biased genomic predictions (Bradford et al., 2019; Pimentel et al., 2022). Despite the possibility of correcting pedigree errors when genomic information is available, this becomes more challenging in U.S. sheep populations. For example, in the largest available dataset, which is comprised of Katahdin sheep, only 10% of the 127,535 animals in the pedigree were genotyped (Pinto et al., 2025b). This limited availability of genotypes restricts the capacity to detect and correct parentage errors, especially in flocks with incomplete or inconsistent pedigree recording.

In this context, the primary objectives of this study were (i) to investigate the effects of pedigree errors, genotyping strategies, and trait heritability on ssGBLUP evaluations of simulated sheep populations, and (ii) identify scenarios that maximize GEBV accuracies and minimize the bias of GEBV prediction. The findings of this study provide guidelines for breeders considering strategies to begin implementing genomic selection in populations with limited or no prior genotyping. Once a sufficiently large and diverse reference population is established, alternative strategies may be more appropriate, particularly those focused on maximizing genetic gain.

## 2 Materials and methods

### 2.1 Population structure

**FIGURE 1:**
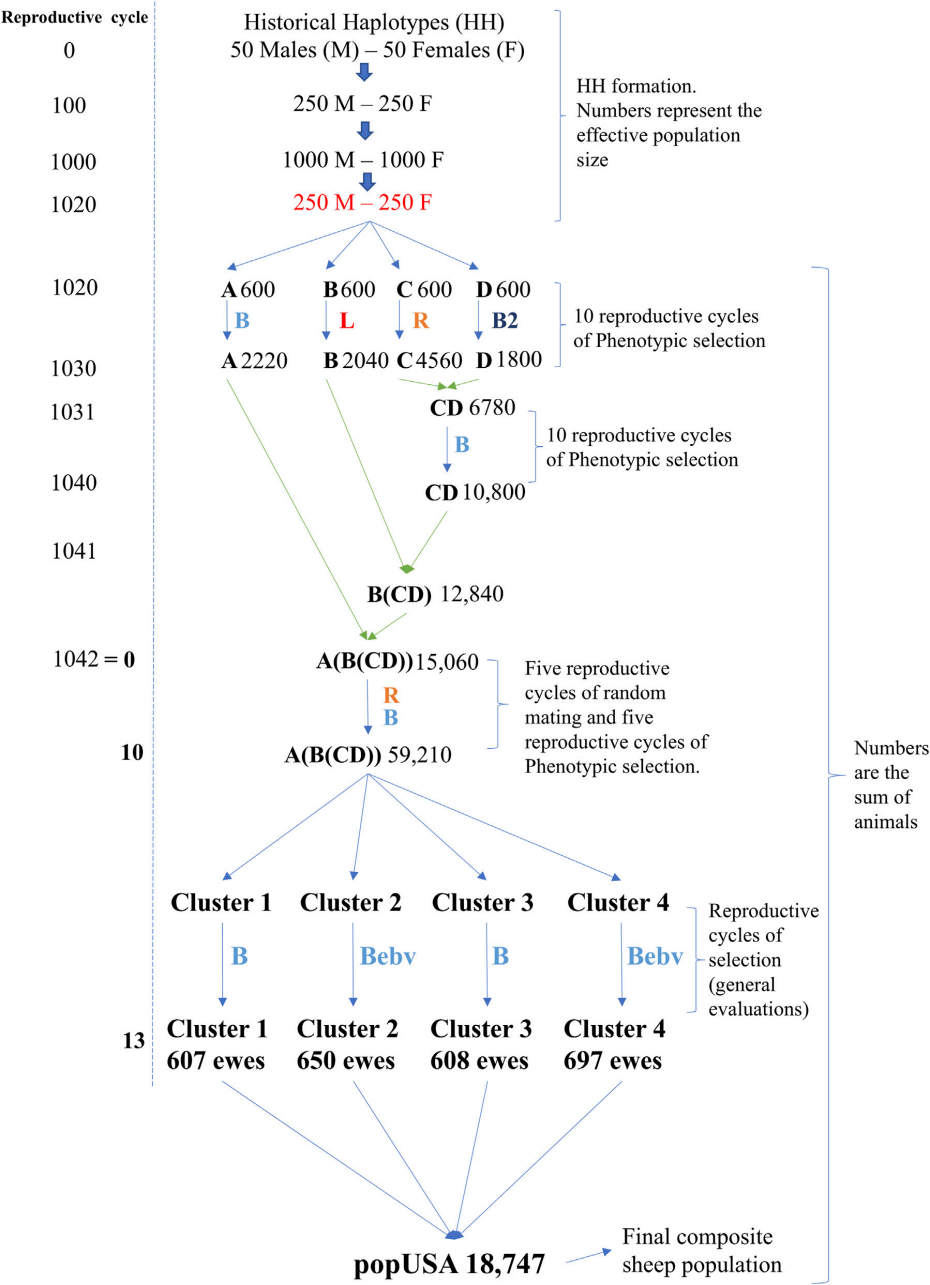
Simulation design to obtain a composite sheep population (popUSA) with flock and cluster structures. Blue-colored B = phenotypic selection to increase the simulated trait; Red-colored L = phenotypic selection to decrease the simulated trait; Orange-colored R = random mating; Dark blue colored B2 = phenotypic selection to increase the secondary simulated trait; Blue colored Bebv = selection based on increased estimated breeding values of the simulated trait. Number on the right side of each population identification are the population sizes.

The simulation mimicked a U.S. composite sheep population (e.g., Katahdin), including its flock connectedness structure (Vargas Jurado et al., 2021; Wilson et al., 2022). This complex design was chosen to reflect the real-world characteristics of the population being simulated. In doing so, it enabled us to provide targeted recommendations for genomic selection strategies that are relevant and applicable to U.S. composite sheep breeds. The R software version 3.4.2 (R. C. Team, 2020) and AlphaSimR package (Gaynor et al., 2021) were used to simulate the founder haplotypes, the formation of a composite breed, and their breeding program. The simulation design is shown in Figure 1. All simulated scenarios were replicated five times with the same structure to form the composite population and its breeding program. Initially, 1,020 generations were simulated to create multiple founder breeds with 300 males (M) and 300 females (F). Although sheep were domesticated more than 10,000 years ago (Deng et al., 2020), the present simulation assumed a sheep population from northern Europe (less than 2000 years ago) as a starting point (Araujo et al., 2021). The first historical haplotypes (generation 0) were simulated with an effective population size (Ne) of 100. A continuous population expansion then occurred leading to Ne of 500 in generation 100 and Ne of 2,000 in generation 1,000, mimicking the demographic expansion observed in European sheep populations over time. After that, a population bottleneck effect was simulated, reducing the historical Ne to 500 in generation 1,020, creating the final historical haplotypes (FHH). The bottleneck, mutation, and genetic drift were the bases for the initial linkage disequilibrium (LD) pattern created. The entire FHH formation was also done under the assumptions of random mating, equal mating opportunity for M and F, and discrete generations, using the backward-in-time simulation process implemented in the Markovian Coalescent Simulator software (MaCS) (Chen et al., 2009) accessed by the function “runMacs2” on AlphaSimR (Gaynor et al., 2021).

Each founder breed had the same initial number of animals (300 M and 300 F with a Ne of 500 from the FHH) and are referred to as breeds A, B, C, and D (Figure 1). From this point forward, all reported values refer to the number of individuals, not the Ne and overlapping generations were assumed. The initial animals for these breeds were generated using the “newPop” internal function from the AlphaSimR package, based on the FHH. Mating took place within breed (i.e., no migration) and replacement animals were selected for ten reproductive cycles (RC). The number of offspring per ewe lambing was set to two lambs, except for the intermediate crosses (F1). Offspring sex was randomly assigned with equal probability for the entire simulation. Two heritability levels, 0.35 and 0.10, were used in the formation of breeds A, B, C, and D. Each heritability level was evaluated separately, and the entire simulation was replicated, applying the same selection and mating strategy described below. The traits were assumed to be normally distributed, with an average of 50 and a standard deviation of 1.23, mimicking an early body weight trait commonly observed once in life in both M and F animals. Only additive genetic effects were simulated as part of the total genetic variance. Different M:F mating ratios and selection criteria were applied to create genetic divergence among the four breeds (Table 1). Breeds A, B, and C were evaluated for the primary trait (trait 1). Breed D, however, was selected based on higher phenotypic values of a different trait. This second trait was used for selection in breed D to further broaden genetic differences among breeds and it had a heritability of 0.18; it was normally distributed with an average of 2.31 and standard deviation of 2.09, and it had a genetic correlation of −0.20 with the primary simulated trait. This secondary trait was used in all scenarios for the RC of breed D only. The final number of animals in each pure breed is shown in Figure 1, reflecting the different demographic and selection parameters applied to each breed to mimic variation in real populations.

**TABLE 1:** Overall simulated pure (A,B,C,D), composite (CD, A (B(CD))) breeds, and intermediate crosses (F1) information.

Simulation parameters	Pure breeds				Intermediate crosses			Composite breeds	
	A	B	C	D	CD F1	B(CD) F1	A (B(CD)) F1	CD	A (B(CD))
Selection criteria^a^	H	L	R	H	R	R	R	H	R and H
Selected trait	1	1	1	2	1	1	1	1	1
Number of reproductive cycles	10	10	10	10	1	1	1	10	10
Male: Female ratio	1:18	1:20	1:22	1:15	1:20	1:18	1:20	1:20	1:14
Proportion of males selected	1.6%	1.3%	3.3%	2%	8%	3.4%	3.5%	0.9%	2.8%
Proportion of females selected	30%	37%	73.3%	30%	100%	100%	100%	17.7%	39.2%

^a^ H, higher phenotypic values; L, lower phenotypic values; R, random.

After establishing divergent pure breeds, a crossbreeding scheme was applied to obtain a composite breed (e.g., Katahdin). The final composite breed had three populations as the starting point: A, B, and CD (e.g., Wiltshire, St. Croix, and Suffolk, respectively). The initial composite CD F1 was generated by random crossing females from breed C and males from breed D (e.g., Norfolk Horn and Southdown, respectively), as shown in Figure 1. After generating a base CD F1 population, ten RC of selection for trait 1 were simulated to establish a new composite breed, as suggested by Rasali et al. (2006). Table 1 and Figure 1 show the details of selection in CD RC and the number of individuals, respectively. Females from this CD population were randomly crossed with males from breed B to generate the B(CD) F1 individuals. The B(CD) F1 females were randomly crossed with males from breed A to generate A (B(CD)) F1 in RC 1042. For each F1 cross, the number of offspring born per ewe was initially set to three to reflect the expected positive impact of hybrid vigor on reproduction compared to the pure and final composite breeds (set at two). To mimic natural variation in litter size, a subset of ewes was then randomly assigned to lose one offspring. Only one F1 population was generated per cross type.

After generating the A (B(CD)) F1, a random selection period was conducted for five RC. Thereafter, this A (B(CD)) composite breed was selected for five additional RC to increase the phenotypic values, as shown in Table 1. This process established the final composite breed with multiple RC and 59,210 animals. Animals from the last three RC of the final composite breed population were used to mimic the structure of the U.S. Katahdin flock. These animals were randomly assigned to one of four clusters and one of four flocks within a cluster (i.e., 16 flocks in total). Figure 1 shows a summary of the cluster level of the four simulated RC in which the number of ewes in each cluster was kept similar: 607, 650, 608, and 697 for clusters 1, 2, 3, and 4, respectively. The selection criteria were also different among clusters. Clusters 1 and 3 selected their animals based on phenotypic values for the simulated trait. Clusters 2 and 4 animals were selected based on EBV for the simulated trait predicted using BLUP. Animals within all clusters were selected to increase phenotypic values or EBV for the same simulated trait.

Variance components, heritabilities, and EBV were re-estimated after each RC for the 16 flocks (4 clusters) because of the selection done in clusters 2 and 4. This process is referred to as the “general evaluation” throughout the paper. We used a pedigree without errors and phenotype information in the BLUPF90+ software (Misztal et al., 2022) for all general evaluations. The true variance components were also obtained using the AlphaSimR package to better compare the variance components pattern among the four RC and the general evaluations (Supplementary Table S1). Three general evaluations were conducted to obtain EBV and apply the cluster-specific selection criteria in the simulated breeding program. An additional (fourth) general evaluation, excluding genomic information, was performed in scenarios where genotyping was based on the highest EBV.

A flock level was also added to each cluster. Each cluster had four flocks of different sizes (number of ewes). Table 2 shows the structure of flocks and clusters. Flocks varied in size from 42 to 375 breeding ewes. By design, the distribution of the flock sizes was skewed toward smaller flocks. The number of breeding sires varied according to the flock size from 3 to 25 and the M:F ratio varied from 1:11 to 1:25 with an average across the entire population of 1:16. A 5% mortality rate was applied at random to breeding ewes within each RC. The ewe culling rate was 15%, and the culling criteria differed based on the cluster to which the flock was assigned. Flocks 1 to 4 (cluster 1) and 9 to 12 (cluster 3) culled their animals based on the lowest phenotypic values. Flocks 5 to 8 (cluster 2) and 13 to 16 (cluster 4) culled their animals based on the lowest EBV for the simulated trait. To simulate a flock effect, a unique constant value was added to each flock’s mean phenotype. These values, randomly drawn from a uniform distribution within the range of 0.10–0.25, ensured variation among the 16 flocks. The same values were added for each flock across all scenarios and replicates. Ewes were kept within a flock for a maximum of 5 RC, while no age-based culling criteria were applied for rams.

**TABLE 2:** Overall simulated flock information per reproductive cycle.

Flock number	Number of breeding ewes	Number of breeding rams	Male: Female ratio	Cluster number	Selection criteria^a^
Flock 01	42	3	1:14	1	Phenotype
Flock 02	170	10	1:17	1	Phenotype
Flock 03	70	5	1:14	1	Phenotype
Flock 04	325	13	1:25	1	Phenotype
Flock 05	45	3	1:15	2	EBV
Flock 06	190	10	1:19	2	EBV
Flock 07	65	5	1:13	2	EBV
Flock 08	350	25	1:14	2	EBV
Flock 09	48	3	1:16	3	Phenotype
Flock 10	200	10	1:20	3	Phenotype
Flock 11	60	4	1:15	3	Phenotype
Flock 12	300	20	1:15	3	Phenotype
Flock 13	51	3	1:17	4	EBV
Flock 14	216	12	1:18	4	EBV
Flock 15	55	5	1:11	4	EBV
Flock 16	375	25	1:15	4	EBV
Total^a^/Average^c^	2562^b^	156^b^	1:16.12^c^		

^a^ Selection criteria EBV: estimated breeding values.

^b^ Values with these superscripts are total values of the population.

^c^ Values with these superscripts are average values of the population.

A mortality rate of 10% was applied randomly to the offspring in every RC before selecting M and F replacements. The replacement rate was 20% to keep the flock sizes constant across RC, and the selection criteria were like the culling criteria. Flocks 1 to 4 (cluster 1) and 9 to 12 (cluster 3) selected replacement animals based on the highest phenotypes. Flocks 5 to 8 (cluster 2) and 13 to 16 (cluster 4) selected replacement animals based on the highest EBV for trait 1. To simulate an RC effect, a small unique constant value (noise) was added to the mean phenotype of each RC, introducing differentiation other than the expected due to selection. These values ranged from 0.01 to 0.05 and were sampled from a uniform distribution.

The genetic connections across flocks mimicked composite sheep populations participating in the U.S. national sheep genetic evaluation (NSIP). Two strategies were used to mimic that structure, one at the cluster level and the other at the flock level. Animals in clusters 1 and 2 selected sires from the same outside flocks in each of the four RC. Animals in clusters 3 and 4 instead selected their outside sires from varying flocks each RC (Table 3). These clusters were designed to represent groups of flocks with similar selection practices and genetic exchange patterns.

**TABLE 3:** Overall simulated flock connectedness among reproductive cycles.

Flock number	Number of breeding ewes	Selection decision	Flocks in which the sires were used (origin of the males)	Purchase of sires strategy
Flock 01	42	1	02 and 09	Fixed
		2	02 and 09	
		3	02 and 09	
Flock 02	170	1	01, 02, and 06	Fixed
		2	01, 02, and 06	
		3	01, 02, and 06	
Flock 03	70	1	03, 04, and 13	Fixed
		2	03, 04, and 13	
		3	03, 04, and 13	
Flock 04	325	1	03, 04, and 17	Fixed
		2	03, 04, and 17	
		3	03, 04, and 17	
Flock 05	45	1	06 and 11	Fixed
		2	06 and 11	
		3	06 and 11	
Flock 06	190	1	06, 08, and 15	Fixed
		2	06, 08, and 15	
		3	06, 08, and 15	
Flock 07	65	1	02, 07, and 16	Fixed
		2	02, 07, and 16	
		3	02, 07, and 16	
Flock 08	350	1	03, 08, and 17	Fixed
		2	03, 08, and 17	
		3	03, 08, and 17	
Flock 09	48	1	09 and 14	Not fixed
		2	09 and 16	
		3	09 and 11	
Flock 10	200	1	10, 12, and 16	Not fixed
		2	04, 08, and 10	
		3	03, 10 and 13	
Flock 11	60	1	10, 11, and 14	Not fixed
		2	11, 12, and 13	
		3	09, 11, and 15	
Flock 12	300	1	10, 08, and 12	Not fixed
		2	11, 12, and 14	
		3	07, 09, and 12	
Flock 13	51	1	10, 13, and 14	Not fixed
		2	09, 13, and 15	
		3	11, 13, and 15	
Flock 14	216	1	04, 14, and 16	Not fixed
		2	04, 12, and 14	
		3	04, 13, and 14	
Flock 15	55	1	04, 14, and 15	Not fixed
		2	08, 13, and 15	
		3	01, 15, and 16	
Flock 16	375	1	08, 14, and 16	Not fixed
		2	02, 15, and 16	
		3	06, 12, and 16	

Selection decision (1, 2, and 3) represents different sire selection strategies used in the simulation. Strategy 1 involved selecting sires after the first general evaluation, strategy 2 after the second general evaluation, and strategy 3 after the third general evaluation.

Table 3 shows the strategies for acquiring sires (sire selection decision) for each flock in all RC. Three categories of flock size were defined: small flocks with up to 50 reproductive ewes, medium flocks with 51–100 ewes, and large flocks with more than 100 ewes per RC. Small flocks acquired 50% of breeding sires from flocks within the same cluster and 50% from flocks in different clusters. Medium flocks acquired 33% of their breeding sires from flocks within the same cluster, another 33% from flocks outside their cluster, and the last 33% from within the flock. Larger flocks acquired 25% of their sires from flocks within the same cluster, another 25% from flocks outside their cluster, and the last 50% from within the flock.

In cases where the number of breeding sires was not evenly divisible for the adopted strategy, the remainder were acquired from outside the flock’s cluster. Another assumption was that some clusters were selected for superior EBV and others for superior phenotypic value. If a flock that selected for superior phenotype acquired a sire from a flock that selected for superior EBV, it would consider only the phenotype, not the EBV, in its selection. Female selection was done only within the flock. These strategies were designed to represent groups of flocks with different sire selection practices and genetic exchange patterns, mimicking the variation in connectedness observed among producer groups (i.e., producers in nearby states) in U.S. composite sheep populations enrolled in NSIP.

The final population used for the genetic prediction analyses was called popUSA (Figure 1), which contained 18,747 animals (2,718 animals for RC 0, plus 3 RC with 5,124 offspring). The Ne of popUSA was simulated to approach 103.7 ± 23.5, which was recently estimated using pedigree information in U.S. Katahdin sheep enrolled in the NSIP (Nilson et al., 2024). All previously determined parameters of the simulations, such as M:F ratios, flock sizes, replacement rates, mortality rates, F1 litter sizes, and connectedness, were adjusted until the targeted Ne was achieved for popUSA. The Ne of the simulated popUSA was calculated based on the increase of realized pedigree inbreeding as proposed by Falconer and Mackay (1996). The average inbreeding used by Falconer and Mackay (1996) formula was calculated based on the outputs from the RENUMF90 software (Misztal et al., 2022).

The average kinship between flocks in the popUSA was calculated using the R package “optiSel” (Wellmann, 2019), based on the pedigree-derived kinship matrix. Pairwise average kinship values were computed between all 16 flocks. Out of all pairwise comparisons, 34 flock pairs had kinship values < 0.002, 62 ranged from 0.002 to 0.005, 19 ranged from 0.005 to 0.01, and 5 exceeded 0.01. These results indicate that the simulated flocks captured a range of genetic connectedness, with most flocks falling in the middle ranges, some being closely related, and others being largely unrelated, mimicking real U.S. sheep populations (Kuehn et al., 2009).

### 2.2 Simulated genome and SNP data

The simulated sheep genome had 26 autosomal chromosomes varying in size from 42,034,648 to 275,406,953 base pairs (total genome length: 2,449,943,362 base pairs) based on the ARS-UI_Ramb_v2.0 reference genome (Davenport et al., 2022). The genome simulation was done simultaneously with the historical haplotype formation using the function “runMACS2” and the software MACS mentioned above. The simulated mutation rate of 2.5 x 10–7 was applied equally across the genome. The number of segregating sites differed for each chromosome, following the same proportion of quantitative trait loci (QTL) relative to chromosome length, plus the number of markers multiplied by a constant (1.2). This strategy mimics a more realistic population genome with more segregating sites than the number of QTL and SNP markers, which is often called an opaque simulator type (Vargas Jurado et al., 2021; Amini et al., 2021). Simulating additional segregating sites yields a more accurate representation of linkage disequilibrium patterns and background variation from neutral and non-causal loci. This enables genomic selection strategies to be evaluated under conditions that closely mimic the polygenic architecture of the simulated traits, thereby making the resulting recommendations more directly applicable to real sheep populations. The total segregation sites were 125,829, varying from 14,145 on chromosome 1 to 2,159 on chromosome 24 (Supplementary Table S2).

A total of 6,800 QTL were simulated along the entire genome. The number of QTL per chromosome varied proportionally to the chromosome size (118 for chromosome 24 to 771 for chromosome 1). The QTL additive genetic effects were sampled from a standard normal distribution and rescaled to match the simulated trait parameters. The number of QTL was based on the AnimalQTLdb release 53 (Hu et al., 2022). To achieve the desired number of SNP after quality control (close to 30,000), and assuming that some QTL were not yet annotated, we increased the number of QTL to 6,800 as mentioned above. In addition, as body weight is a highly polygenic trait, we assumed that many causal variants remain undetected in sheep populations and, therefore, modeled a larger number of QTL to better approximate the trait’s underlying genetic architecture. The number of markers (bi-allelic SNP) was also proportional to the chromosome length. The number of SNP ranged from 1,681 (chromosome 24) to 11,016 (chromosome 1), totaling 97,998 SNP (Supplementary Table S2). Quality control methods were applied using the default parameters of the BLUPF90+ software (Misztal et al., 2022). Specifically, SNP with a minor allele frequency (MAF) < 0.05 were removed. A total of 34,246 ± 947 SNP were available for the analyses, which is comparable to the number of SNP in real U.S. sheep genomic evaluations (Araujo et al., 2023).

### 2.3 Prediction of (G)EBV and dataset partitioning

The genetic and genomic predictions in popUSA were performed in the BLUPF90+ software (Misztal et al., 2022). The AI-REML algorithm was used for all scenarios and replicates to estimate variance components. The model used in both methods was:

y=Xb+Zu+e

where **y** is the vector of phenotypic records, **b** is the vector of fixed effects (flock-cluster, RC, and sex), **u** is the vector of direct additive genetic effects with $u\;∼\;N\left(0,H\sigma_{g}^{2}\right)$ in the ssGBLUP approach and $u\;∼\;N\left(0,A\sigma_{g}^{2}\right)$ in the pedigree-based BLUP approach, **e** is the vector of random errors with $e\;∼\;N\left(0,I\sigma_{e}^{2}\right)$ , and **X** and **Z** are the incidence matrices for the fixed and additive genetic effects, respectively. The inverse of **H** was computed as described by Aguilar et al. (2010) and the default values for weighting parameters from the BLUPF90+ software (Misztal et al., 2022).

The popUSA was divided into training and validation sets to assess the observed and accuracy, bias, and dispersion of GEBV predictions. The training sets were composed of individuals from the first to third RC, and the validation set included animals from the fourth RC. The number of genotyped individuals in the training and validation sets varied according to the evaluated scenarios, which will be explained in the following sections. In a scenario with 100% of animals being genotyped, 13,623 animals in the training set had phenotypic, pedigree, and genomic information and 5,124 animals in the validation set had only genomic and pedigree information. The number of animals in the pedigree data for all scenarios and replicates was 21,027, which includes the 18,747 animals in the popUSA plus the parents of sampled individuals, who were included as founders.

### 2.4 Evaluated scenarios

A total of 6,144 unique scenarios were evaluated, with the popUSA divided into training and validation sets (Supplementary Figure S1). As previously mentioned, two trait heritability levels (0.35 and 0.10) were simulated. Furthermore, three genotyping strategies were evaluated: choosing the animals to be genotyped based on the highest phenotypes (Phenotype), highest BLUP EBV (EBV), or at random (Random).

Different proportions of misidentified sires were simulated, 0, 5, 10, and 20%, to evaluate the impact of pedigree errors (misidentified sires and missing pedigree). To simulate misidentified sires, we randomly replaced sire IDs in the pedigree while ensuring that a given sire ID could be assigned to multiple offspring. This mimics real-world errors in which all lambs in a litter can possibly share the same incorrect sire, with the assigned sire possible drawn from within the flock. The algorithm maintained realistic pedigree structures by preventing impossible sire-offspring relationships. After applying the proportion of misidentified sires, different proportions of missing pedigree information were also simulated: 0, 5, 10, and 20% (Supplementary Figure S1). The total proportion of missing pedigree was equally split between sires and dams. Overlap between both pedigree error types was not allowed. No correction was applied to restore the true parentage after introducing pedigree errors. That is, misidentified sires remained uncorrected throughout all scenarios to reflect the impact of unrecognized errors in practice.

Different proportions of genotyped animals for each sex were also evaluated. All possible combinations of 0, 5, 10, 15, 20, 40, 80, and 100% of genotyped females or males were compared (Supplementary Figure S1). All proportions of genotyped animals were applied considering sex and RC levels (1, 2, and 3) to avoid misrepresenting certain RC in the training dataset. For example, if a scenario included 20% of males and 40% of females genotyped, these proportions were applied separately within each RC level (Supplementary Table S3).

### 2.5 Scenario comparison

The (G)EBV were estimated in each validation set for all scenarios. The true accuracy, bias, and dispersion (acc, bias, and disp, respectively) were then estimated on the validation set for each scenario using the true breeding values (TBV). The validation statistics obtained using the TBV were calculated as:

$$acc=\frac{cov\left(u,\hat{u}\right)}{\sqrt{var\left(u\right)var\left(\hat{u}\right)\;}}$$

$$bias=\bar{u}-\bar{\hat{u}}$$

disp=covu,u^varu^

where $cov\left(u,\hat{u}\right)$ is the covariance between TBV ( u ) and (G)EBV ( $\hat{u}$ ), $var\left(u\right)$ and $var\left(\hat{u}\right)$ are the variance of TBV and (G)EBV, respectively, $\bar{u}$ and $\bar{\hat{u}}$ are the averages of TBV and (G)EBV, respectively, all from the validation sets.

Following Bermann et al. (2024), deviations from 1 (regression slope only) were considered as the dispersion values. Dispersion closer to 1 is desirable because it indicates GEBV variance matches TBV variance, with no inflation or deflation. All statistics were compared to their respective scenario without genomic information yet with a perfect pedigree to assess the impact of including genomic information and pedigree errors in the analyses. In other words, the percentage change or difference was calculated for each parameter, comparing the average value of the evaluated ssGBLUP scenarios against the BLUP with a perfect pedigree (BLUPPP or baseline) (Vickers, 2001). For instance, the acc percentage change was calculated as:

$$acc\;percentage\;change=\frac{{acc}_{ssGBLUP}-{acc}_{BLUPPP}}{{acc}_{BLUPPP}}\times 100$$

where ${acc}_{ssGBLUP}$ is the average acc value for the evaluated scenario, ${acc}_{BLUPPP}$ is the acc value for BLUPPP respective scenario.

The same percentage change, was applied to all statistics, totaling six parameters for each scenario comparison (for a more detailed description, see Supplementary Table S4). Pearson correlation coefficients were calculated between acc, bias, and disp to evaluate the average association of these metrics among all scenarios. A linear model was used to estimate the effect of each predictor (scenarios) on each response variable (acc, bias, disp, and percentage change for each). Each response variable were analyzed using linear models that included fixed effects of heritability (0.10 or 0.35), proportion of misidentified sires (0, 5, 10, or 20%), proportion of missing pedigree (0, 5, 10, or 20%), genotyping strategy (Phenotype, EBV, or Random), proportion of males genotyped (0, 5, 10, 15, 20, 40, 80, or 100%), and proportion of females genotyped (0, 5, 10, 15, 20, 40, 80, or 100). The model accounted for all two-way through six-way interactions among these factors, as their combined effects were of interest. The random residual error term was assumed to be normally distributed with mean zero and homogeneous, independent variances. Pairwise comparisons of means for fixed effect levels were made when the analyses of variance indicated a significant effect (*P* < 0.05), with Tukey’s test at a 5% significance level. The “emmeans” (Lenth et al., 2021) and “agricolae” (De Mendiburu and Simon, 2015) R packages were used for the comparison of the means (R. C. Team, 2020).

## 3 Results

### 3.1 Accuracy, bias, and dispersion of genomic predictions

The estimated variance components closely matched the true values across replicates and scenarios for both simulated heritability levels. Both additive genetic and residual variances followed patterns consistent with the true values, showing only minor fluctuations across general evaluations. Heritability estimates initially deviated from the true values, slightly overestimated for the moderately heritable trait and underestimated for the lowly heritable trait, but gradually converged as evaluations progressed. From the third general evaluation onward, estimates stabilized and remained consistent, even with the addition of genomic information. It is important to note that these results relate specifically to the fourth RC of the popUSA.

The main effect of all evaluated scenarios impacted (*P* < 0.005) all parameters. For acc and its percentage change, up to 4-way interactions were significant. All other parameters exhibited significant interactions up to 5-way interaction levels. These results indicate that the accuracy, bias, and dispersion of genomic predictions are influenced by complex combinations of factors rather than any single factor alone, highlighting the need for integrated strategies when designing genomic selection programs. The following sections focus on the most important interaction levels, presented using least squares means (marginal means). While higher-order interactions were significant, they did not invalidate inferences drawn from lower-order interactions. This approach facilitated a clearer understanding of complex interactions by emphasizing combinations with the most practical implications for sheep breeding programs and genomic predictions, thereby aiding the interpretation and application of the findings.

Correlation coefficients between different parameters were similar for both simulated heritabilities (Table 4). When accuracy increased, bias decreased, and dispersion either decreased, going towards 1 (less overdispersion), or increased, going towards greater than 1 (under-dispersion). When bias increased, (G)EBV became more over-dispersed.

**TABLE 4:** Pearson correlation coefficients (SE) between true parameters for heritability level of 0.35 (above diagonal elements) and 0.10 (below diagonal elements).

Parameter	acc	bias	disp
acc	1	−0.25 (0.005)	0.85 (0.007)
bias	−0.32 (0.005)	1	−0.28 (0.007)
disp	0.76 (0.006)	−0.37 (0.006)	1

Parameter: acc, prediction accuracy; bias, prediction bias; disp, prediction dispersion.

### 3.2 Genotyping strategies scenarios

Prediction accuracies were higher with the random genotyping strategy for both moderate and low heritability traits. Table 5 shows the accuracy metrics and their percentage change by genotyping strategy for the simulated heritability level of 0.35. On average, the Random strategy had greater acc by 5.58% ± 0.31% when compared to the BLUPPP scenario (baseline is 0.52). However, on average, the acc decreased by 2.97% ± 0.36% in the Phenotype genotyping strategy and by 4.74% ± 0.39% in the EBV genotyping strategy. With few exceptions, the trends observed for accuracy for the lowly heritable trait were like the moderately heritable trait although, as expected, with smaller values (Table 5).

**TABLE 5:** Average true accurary, bias, and, dispersion of genomic prediction with their respective percent change (%) for a trait with a heritability level of 0.35 and 0.10 by genotyping strategy.

Parameter	Baseline value	Heritability	Genotyping strategy		
			Random	Phenotype	EBV
acc	—	0.35	0.55_a_ ± 0.001	0.50_b_ ± 0.001	0.49_c_ ± 0.002
		0.10	0.48_a_ ± 0.001	0.42_b_ ± 0.001	0.41_c_ ± 0.001
bias	—	0.35	0.36_c_ ± 0.003	1.26_a_ ± 0.011	1.10 _b_ ± 0.009
		0.10	0.21_c_ ± 0.002	1.21_a_ ± 0.014	1.09 _b_ ± 0.012
disp	—	0.35	0.85_a_ ± 0.001	0.73_c_ ± 0.002	0.74_b_ ± 0.002
		0.10	0.91_a_ ± 0.001	0.70_b_ ± 0.003	0.71_b_ ± 0.004
acc change	0.518	0.35	5.58_a_ ± 0.31	−2.97_b_ ± 0.36	−4.74_c_ ± 0.39
	0.441	0.10	8.58_a_ ± 0.28	−5.28_b_ ± 0.38	−8.22_c_ ± 0.42
bias change	1.358	0.35	−73.55_c_ ± 0.28	−7.44_a_ ± 0.80	−18.73_b_ ± 0.67
	1.041	0.10	−79.23_c_ ± 0.23	−16.48_a_ ± 1.34	6.04_b_ ± 1.15
disp change	0.941	0.35	−9.81_a_ ± 0.15	−22.89_c_ ± 0.23	−21.55_b_ ± 0.24
	0.953	0.10	−4.84_a_ ± 0.15	−26.27_b_ ± 0.33	−25.82_b_ ± 0.36

Parameter: acc, prediction accuracy; bias, prediction bias; disp, prediction dispersion.

Random, randomly genotyping; Phenotype, genotyping superior phenotypes, and EBV, genotyping superior EBV., within each row, genotyping strategies with no common suberscript are different (*P* < 0.05).

Similar trends were also observed between the moderate and lowly heritable traits for the bias results. For a heritability of 0.35, the bias values were smaller when compared to the BLUPPP (baseline of 1.358), varying from −73.55% ± 0.28% for the Random strategy to −7.44% ± 0.80% for the Phenotype strategy (Table 5). However, for the lowly heritable trait, bias values were greater than their respective baselines (1.041 for the bias) for the EBV strategy (Table 5).

On average, the scenarios were over-dispersed across both heritability levels. The percent disp changes were negative across both heritability levels and all genotyping scenarios (Table 5). This indicates even greater overdispersion compared to the BLUPPP scenario (baseline). The baseline disp values were 0.94 for a heritability of 0.35 and 0.95 for a heritability of 0.10.

### 3.3 Pedigree scenarios

The impacts of pedigree errors on parameters were similar for both heritability levels. Generally, as the pedigree errors—both misidentified sires (MS) and missing information (MI)—increased, the average accuracy decreased, bias increased, and dispersion either increased towards under-dispersion (greater than 1) or decreased towards more overdispersion (lower than 1). Therefore, for simplicity, only the results for the 0.35 heritability level are presented.

**FIGURE 2:**
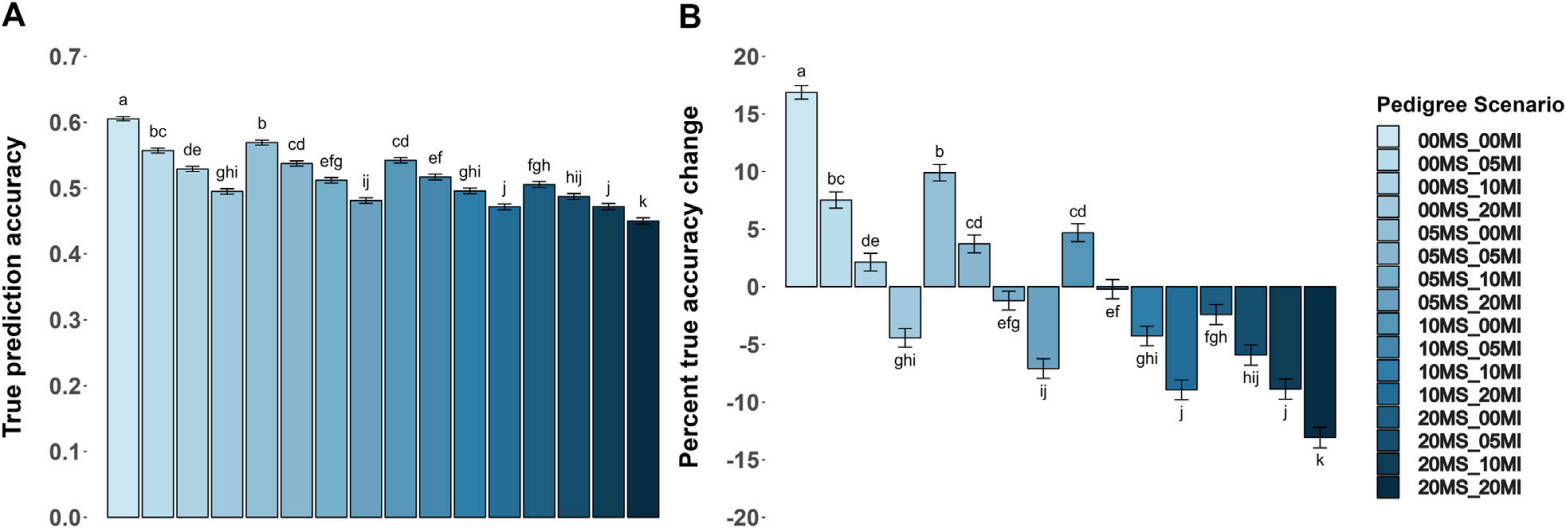
Average true accuracy of genomic prediction with their respective percentage change for a trait with a heritability level of 0.35 by pedigree error scenarios. Misidentified sire (MS) and missing information (MI) were each evaluated at proportions of 0, 0.05, 0.10, and 0.20. **(A,B)** Show the true accuracy results and the percentage change compared to the baseline (BLUPPP) scenario for the true accuracy, respectively. Baseline value is 0.518 in **(B)**. Within each panel, genotyping strategies with no common superscript are different (*P* < 0.05).

Prediction accuracies were greatest (*P* < 0.05) in the scenarios with no MS and MI. Figure 2 shows the accuracy metrics and their percentage change by pedigree error scenario for the heritability level of 0.35. The only scenario where the acc achieved values greater than 0.60 was without errors (Figure 2A). Also, as the proportions of MS and MI increased, the acc decreased, with the MI having a greater impact on reducing the acc. In all scenarios where the proportion of MI and MS were opposite (e.g., 5% of MS and 0% of MI versus 0% of MS and 5% of MI), the MI effect had a greater numerical (P > 0.05) impact on reducing the acc. As pedigree errors increased, accuracy decreased.

On average, genomic information increased acc in a scenario without pedigree errors by 17% compared to their BLUPPP scenario (the baseline acc was 0.518), as shown in Figure 2B. Scenarios where one or both MI and MS was 5% or less, and the scenario with 10% of MS and 0% of MI, had an average acc greater than the baseline. All other scenarios could not overcome the losses due to pedigree errors by including genomic information.

For all scenarios the predictions of breeding value were biased downwards (greater than 0). The smallest bias (±SE) value (0.39 ± 0.09) was in the scenario with 0% MS and 0% MI (Supplementary Figure S2a). As with the results on accuracy, MI had a greater impact on bias than MS. In all scenarios where the proportion of MI and MS were opposite (e.g., 5% of MS and 0% of MI versus 0% of MS and 5% of MI) the MI effect had a greater impact in increasing (*P* < 0.05) the bias. When 20% of both pedigree error types were evaluated, the bias was greatest at 1.70 ± 0.15.

Except for two pedigree scenarios (10% MS and 20% MI; 20% MS and 20% MI), incorporating genomic information was able to reduce the bias compared to the baseline (1.358), on average. The greatest reduction (*P* < 0.05) from the others (±SE) was −71.28% ± 0.97 in the scenario without pedigree errors, and the smallest reduction was −9.87% ± 1.29 in 20% MS and 10% MI scenarios (Supplementary Figure S2b).

Overall, all values of the disp were over dispersed (lower than 1), and the extent of overdispersion increased as the proportions of pedigree errors increased. In the scenario without pedigree errors, inclusion of genomic information did not affect the statistic for dispersion compared to the baseline (0.941 for disp in the BLUPPP (Supplementary Figure S3b). Additionally, as the proportion of pedigree errors increased, the reduction in the parameter values clearly showed greater overdispersion.

### 3.4 Genotyping proportion scenarios

As in the pedigree error scenarios, both trait heritabilities show similar trends. Additionally, the Phenotype-based genotyping strategy had similar results as the EBV strategy. Generally, as the proportion of genotyped animals increased, average accuracy increased, bias decreased, and dispersion approached 1. Additionally, with more pedigree errors accuracy decreased while bias and dispersion increased. Therefore, for simplicity, only results for the trait with moderate heritability, and the Random and EBV genotyping strategies are presented. These results will be shown without pedigree errors or with 20% MI and 20% MS, which represent the extreme scenarios for all statistics.

**FIGURE 3:**
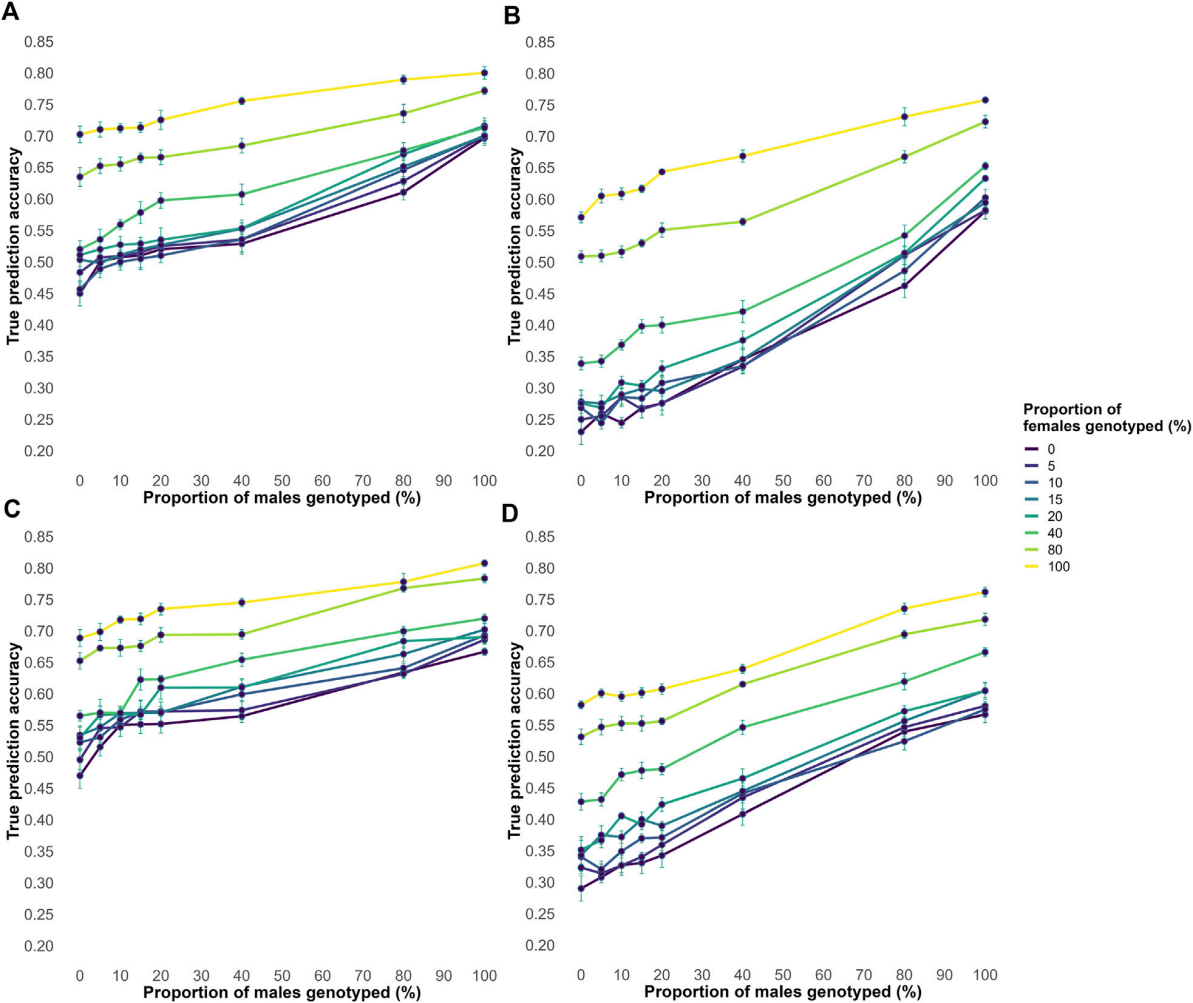
The average true accuracy of genomic predictions in simulations for a trait with a heritability level of 0.35 for increasing proportions of male and female animals genotyped. Interaction between proportions of males and females genotyped, all possible combinations of 0, 5, 10, 15, 20, 40, 80, and 100% in both factors are shown. **(A)** Shows the true accuracy results for the EBV genotyping criteria without pedigree errors. **(B)** Shows the true accuracy results for the EBV genotyping criteria with 20% of misidentified sires and 20% of missing information. **(C)** Shows the true accuracy results for the Random genotyping criteria without pedigree errors. **(D)** Shows the true accuracy results for the random genotyping criteria, with 20% misidentified sires and 20% missing information.

Across all male and female genotyping proportions, acc increased as the proportion of genotyped animals increased (Figure 3). In both the EBV and Random genotyping strategies, accuracies were highest when all animals were genotyped and lowest when no animals were genotyped. Pedigree errors reduced accuracy across all scenarios, but the overall ranking of the evaluated strategies remained the same. Notably, accuracy under the Random genotyping strategy was generally higher than under the EBV strategy, even when pedigree errors were present.

Unlike accuracy, bias trends differed between the selective and Random genotyping strategies (Supplementary Figure S4). In selective genotyping, bias increased until 40% of both sexes were genotyped before decreasing when at least 80% of one sex was genotyped. The highest biases occurred in selective genotyping, particularly with pedigree errors. In contrast, bias under Random genotyping steadily declined as more animals were genotyped, though pedigree errors led to slightly higher bias.

The disp results followed a similar trend in all scenarios. As the proportion of genotyped animals increased, the dispersion value increased towards 1, meaning less overdispersion (Supplementary Figure S5). Furthermore, when all animals were genotyped, the dispersion values were closest to 1 in all scenarios. For the scenario with Random genotyping without pedigree errors, dispersion values showed no clear pattern up to 20% genotyped, after which the various scenarios behaved similarly. In all scenarios other than Random genotyping without pedigree errors, dispersion values diverged (P < 0.05) when at least 40% of one sex was genotyped. In the scenario with a Random genotyping strategy and no pedigree errors (Supplementary Figure S5c) the dispersion values were closer to 1 as compared to the other scenarios.

### 3.5 Genotyping proportion, pedigree, and sex scenarios

**FIGURE 4:**
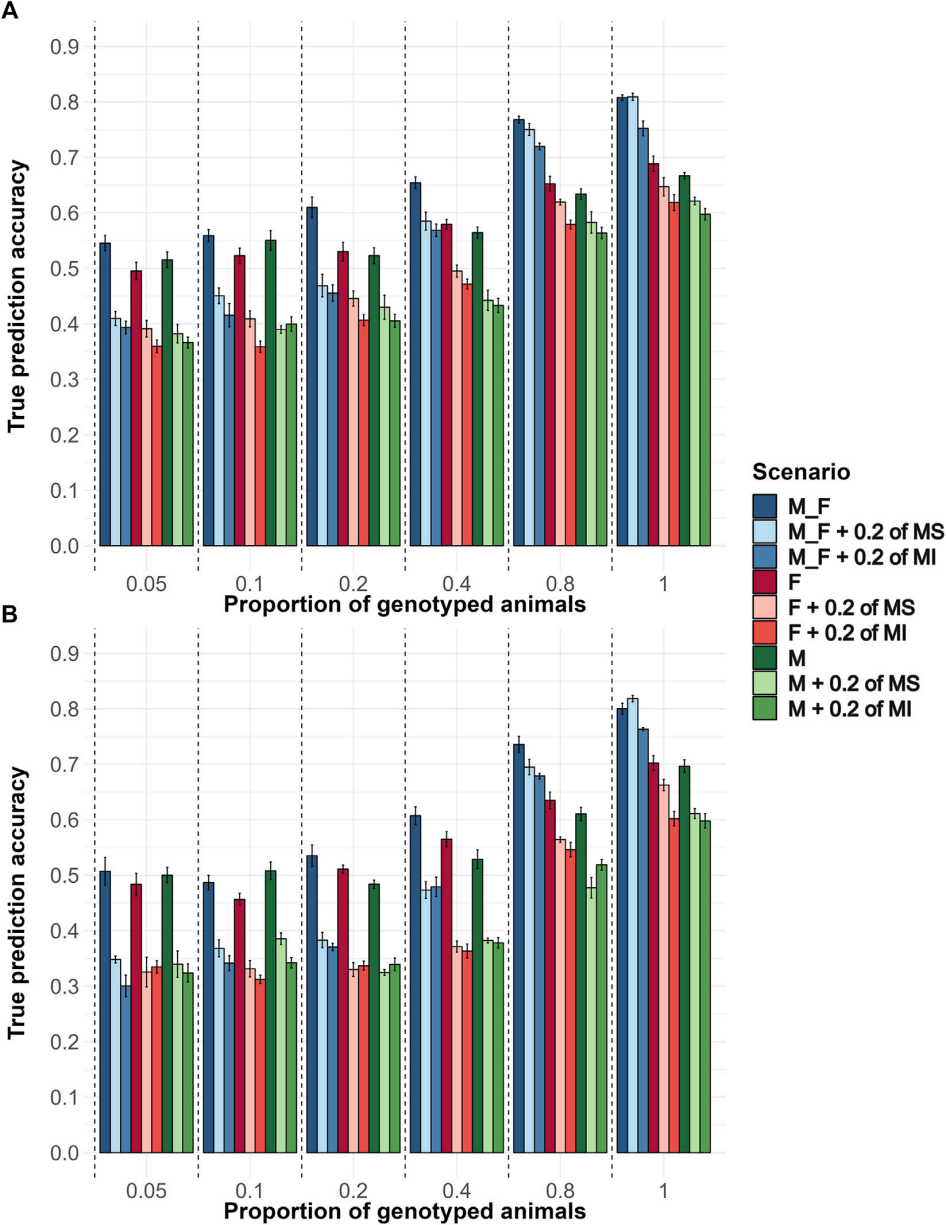
The average true accuracy of genomic predictions in simulations for a trait with a heritability level of 0.35 by Genotyping Proportions and Pedigree Scenarios. Interaction between proportions of males (M), females (F), and males and females (M_F) genotyped, with scenarios without pedigree errors, with 20% of misidentified sires (MS) or with 20% of missing information (MI). Combinations of 5, 10, 20, 40, 80, and 100% of both males and females were genotyped, and the same proportions were applied for each sex separately. **(A)** Shows the true accuracy results for the Random genotyping criteria, and **(B)** Shows the true accuracy results for the EBV genotyping criteria.

In the genotyping proportion scenarios described above, the extent of pedigree errors evaluated was 20% MS and 20% MI. To better understand the impact of each type of pedigree error, we tested 20% MS and 20% MI separately, along with the sexes and proportions of the animals genotyped. Generally, the average accuracy increased as the proportion of genotyped animals increased. MI had a greater impact in reducing the prediction accuracies than MS. Genotyping the same proportion of animals from each sex yielded significantly higher accuracies than genotyping only males or only females. When only a small proportion (up to 10%) of animals was genotyped, prioritizing genotyping males resulted in slightly greater accuracies. However, when a higher proportion of animals were genotyped, genotyping females resulted in greater accuracies. When comparing the Random (Figure 4A) with the EBV (Figure 4B) genotyping strategy in a pairwise way for the same scenario, the Random strategy had on average accuracies that were 0.05 higher. Also, as the proportion of genotyped animals increased, the impact of both pedigree error types decreased, resulting in accuracy values closer to their respective scenarios without pedigree errors.

Average bias decreased as the proportion of genotyped animals increased (Supplementary Figure S6). When randomly genotyping only males with or without pedigree errors, the bias values were higher than other interaction levels within and across genotyping proportions. As the proportion of genotyped animals increased, bias decreased in most scenarios, especially in those with missing pedigree information, emphasizing the greater impact of missing pedigree information on bias. In all scenarios, regardless of pedigree error type and whether both sexes or only males or females were genotyped, the bias of prediction increased as the proportion of genotyped animals rose to 40%. Bias began to decrease when high proportions of animals (80%) were genotyped.

The average dispersion levels were lower than 1, demonstrating inflation of GEBV (Supplementary Figure S7). When the random genotyping strategy was used, dispersion values were statistically closer to 1 compared to their respective scenarios under the EBV-based genotyping strategy. Also, when no pedigree errors were introduced, and M and F were genotyped, the dispersion values were no lower than 0.9 in the Random genotyping strategy and no lower than 0.76 in the EBV strategy. However, when any proportion of pedigree error was considered, the dispersion value dropped substantially, achieving values closer to 0.52 when fewer animals of one sex were genotyped in the EBV strategy. A similar but smaller drop was also observed in the Random strategy.

## 4 Discussion

Across all RC, the genetic parameters and their respective estimates remained relatively consistent. In agreement with theoretical expectations for populations under selection, the popUSA achieved a certain Bulmer equilibrium. This equilibrium was reflected in the stabilization of the additive genetic variance during the third general evaluation. This genetic evaluation incorporated information from exactly four RC of the breeding program and a genetic background spanning the previous RC of the composite breed [A (B(CD))]. These findings align with the theoretical work of Bulmer (1971), Dekkers (1992), Bijma and Van Arendonk (1998), and Gorjanc et al. (2015), meaning that the gametic-phase disequilibrium caused by selection was stabilized. General evaluations and variance component estimation were performed using a perfect pedigree and phenotypic records. At the third general evaluation, a pedigree depth of up to six generations (calculated with the R package 'optiSel' [Wellmann, 2019]) was sufficient for estimating the variance components of the popUSA founders. Slight changes in variance component estimates in the “Fourth (G)” general evaluation were caused by incorporating genomic information and pedigree errors in the analyses. The values of this final general evaluation were used in the predictions, and their slight differences are reflected in the validation statistics and accounted for in the five replicates.

### 4.1 Accuracy, bias, and dispersion of genomic predictions

While direct cause-and-effect relationship cannot be inferred, the correlation results between metrics revealed an interesting trend. As the accuracies increased, the dispersion values of GEBV tended to deflate, moving closer to 1. In a study investigating the inflation of GEBV in Holstein cows, Misztal (2017) also found decreased inflation and increased accuracy as the numerator relationship matrix became more complete, like what Westell et al. (1988) found. In Misztal (2017) analyses, they accounted for inbreeding and unknown parent groups. Another way of reducing inflation, if inbreeding is not accounted for, is by testing different values for τ and ω while building the $H^{-1}$ matrix, as Harris et al. (2012) and Tsuruta et al. (2013) suggested. However, more recently, Nilforooshan (2022) concluded that altering these τ and ω is not recommended due to recent ssGBLUP improvements, such as accounting for inbreeding. Consequently, the present study did not evaluate the impact of changing values of *τ* and *ω* since accounting for inbreeding is a default procedure in recent BLUPF90+ software (Misztal et al., 2022). A value of 1 was assumed for both τ and ω while α and β values were 0.95 and 0.05, respectively, in all scenarios.

This study focused on validation statistics rather than long-term measures such as genetic gain. The aim was to assess the immediate impact of incorporating genomic information into evaluations, not the cumulative response over successive reproductive cycles. Genetic gain is a downstream outcome of prediction quality, while accuracy, bias, and dispersion provide direct and cycle-specific (validation animals) measures of how well genomic information improves evaluation (Boichard et al., 2016; Bermann et al., 2021). These metrics are also the established standard in genomic validation studies prior to adoption of genomic selection in new populations (Legarra and Reverter, 2018), ensuring our results remain directly interpretable and broadly comparable across studies. This focus is also consistent with the objectives of our study, which considered strategies for establishing a new reference population in otherwise ungenotyped flocks. Once a sufficiently large and diverse reference population is established, realized genetic gain becomes a more relevant criterion for comparing strategies.

### 4.2 Genotyping strategies scenarios

For both simulated trait heritability levels, the Random genotyping strategy yielded greater accuracy, less biased predictions, and less inflated GEBV than any selective genotyping strategy. Similar results have been reported in simulation studies comparing genotyping strategies for building reference populations in a ssGBLUP framework in beef cattle (Esrafili Taze Kand Mohammaddiyeh et al., 2023), pigs (Liu et al., 2023), and a general species that was not litter bearing (Boligon et al., 2012). Not choosing animals to be genotyped based on pre-determined criteria results in a more informative (diverse) and unbiased reference population, which can improve the accuracy of genomic predictions. The advantage of randomly genotyping animals is even more pronounced in composite sheep populations, which have relatively higher Ne compared to purebred sheep and other livestock species (Nilson et al., 2024; Oliveira et al., 2020). Populations with higher Ne need larger reference populations to achieve similar GEBV prediction accuracies for a given trait heritability (Daetwyler et al., 2012). By randomly choosing animals for genotyping, the number of animals needed to achieve the same prediction accuracy was reduced, which would lead to a significant reduction in genotyping costs over time (more details in the following discussion sections). This is substantiated by evaluating percentage changes from baseline, where only the random genotyping achieved higher accuracy and reduced bias.

These findings have particular relevance for sheep breeding programs, which typically operate with smaller flock sizes, lower use of artificial insemination, and reduced pedigree connectedness compared to cattle or pigs. These characteristics amplify the consequences of pedigree errors and increase the reliance on well-designed genotyping strategies, making random genotyping especially valuable in sheep populations.

Several studies have compared the impact of genotyping animals with extreme phenotypes or EBV (superior and inferior animals) or just the inferior animals (Ødegård and Meuwissen, 2014; Perez et al., 2019). These selective genotyping strategies for the extremes may result in greater GEBV accuracies but their bias and dispersion were also greater compared to random genotyping. Gowane et al. (2019) stated that, in practice, it is common for producers to prioritize genotyping animals with superior EBV or phenotypes. For these reasons, we did not test additional scenarios that genotyped only genetically inferior animals (bottom rankings) or both extremes. While more robust genotyping strategies, such as optimizing for family structure, relatedness, or population representativity, have been proposed (Pszczola et al., 2012; Rincent et al., 2012), they often require detailed pedigree and complete data not available in many U.S. flocks. This could confound results under pedigree error scenarios. Additionally, we did not include these strategies to avoid further increasing the number of simulated scenarios, which were already substantial due to the combination of genotyping strategies, pedigree error types, and heritability levels.

To our knowledge, this is the first study to jointly investigate genotyping strategies alongside pedigree errors and varying heritabilities in a simulated sheep population, providing a comprehensive evaluation relevant to practical implementation. The results presented here represent marginal averages for each genotyping strategy, accounting for pedigree errors and all genotyping proportions. This explains the greater inflation of GEBV for the youngest animals (reflected in smaller dispersion values) across all strategies compared to their baselines (Supplementary Figure S4c: Additional File 3).

### 4.3 Pedigree scenarios

Our results showed the negative impact of pedigree errors (MI or MS) on (G)EBV accuracy, bias, and dispersion even when genomic data were included in the analyses. Parentage misidentification has been a concern since the early stages of genetic evaluations (Van Vleck, 1970). Even with relatively low levels of pedigree errors, the accuracy of additive genetic relationships among animals is reduced, with estimates of variance components and the accuracy of predictions negatively impacted, reducing genetic progress. The numerically (P > 0.05) greater negative impact of MI compared to MS was unexpected, but it may be because MI breaks pedigree links entirely, potentially leading to underestimated relationships and reduced connectedness based on the **A** matrix. This could create a stronger structural mismatch with the **G** matrix than MS, for which pedigree paths remain present, though incorrect, and can be partially accounted for by **G** when building the **H** inverse. As a result, MI may have resulted in greater GEBV bias and dispersion. However, it is important to highlight that both types of pedigree errors should be avoided in breeding programs.

Pimentel et al. (2024), like our findings, found negative impacts of pedigree errors in a recent study evaluating simulated dairy cattle populations. As the proportions of MS increased, the acc decreased. However, in their study the bias was not impacted by pedigree errors, and the disp levels increased from less than 1 towards greater than 1 (deflation of GEBV).

As previously mentioned, potential mismatch between $A_{22}$ and G are known to cause bias, deflation, or inflation of predicted (G)EBV (Misztal, 2017). We further confirmed this mismatch by examining the correlation between the off-diagonal elements of $A_{22}$ and G , which declined from 0.89 with standard deviation on 0.02 (in the scenario without pedigree errors and with 100% genotyped animals) to 0.44 with standard deviation on 0.04 (in the scenario with 20% MS and all animals genotyped). Comparing the scenario without pedigree errors to the baseline, the inclusion of genomic information resulted in higher GEBV accuracies, reduced bias, and similar inflation for the parameters. Similar results were observed for a composite sheep simulation study for heritability levels of both 0.30 and 0.10 (Araujo et al., 2021). These results confirm the hypothesis that pedigree mismatching can cause inflation or deflation. Additionally, the current study did not test unknown parent groups or metafounders to account for missing pedigree information. The impact of these approaches in U.S. composite sheep breeds needs to be further investigated, as they have been shown to yield more accurate and less biased genomic predictions (Legarra et al., 2015; Koivula et al., 2022; Macedo et al., 2020).

Several studies reported that a proportion of animals suffered incorrect parentage: between 5% and 15% in Danish dairy cattle (Christensen et al., 1982), between 2.9% and 5.2% in the Israeli Holstein population (Ron et al., 1996), 10% in UK Holstein-Friesian dairy cattle population (Visscher et al., 2002), and more recently in a sheep population, 5%–6% in UK Texel sheep (Kaseja et al., 2022). We calculated the proportion of pedigree errors within NSIP Katahdin sheep using the SeekParentF90 software (Misztal et al., 2022). Across 10,032 animals with pedigree and genomic information, the proportion of pedigree mismatches was 1.6% for dam-offspring, 8.0% sire-offspring, and 5.5% for sire and dam-offspring. However, these errors might be higher in practice as producers use genomic information to correct pedigrees. These findings, along with simulation results, highlight that uncorrected pedigree errors in NSIP data can significantly reduce the accuracy of (G)EBV, particularly for young animals. Furthermore, such errors can increase bias and cause inflation or deflation in EBV, potentially leading to suboptimal selection decisions. Addressing these errors is critical to ensure the reliability of genetic evaluations and improve the overall effectiveness of breeding programs. Furthermore, using a reference population with genomic information through the ssGBLUP method proved to be a suitable approach for addressing existing pedigree errors. On average, this method performed well in scenarios with up to 5% MS and 5% MI, or 10% MS alone. In these scenarios, ssGBLUP achieved greater accuracy than the baseline, reduced bias, and showed a slight decrease in disp.

In this study, we intentionally did not correct misidentified sires by replacing them with the true sires. This approach was chosen to mimic the reality of many livestock populations, where parentage errors are often undetected or unresolved due to limited genotyping coverage. However, given the scenarios compared, correcting pedigrees based on genotype information would potentially bias the comparisons. Where the percentage of animals genotyped was higher, more pedigree errors would be detected and corrected. We therefore disabled BLUPF90’s default behavior of removing progeny with genotype conflicts, ensuring that all animals, including those with detectable errors, were retained in the analysis.

### 4.4 Genotyping proportion scenarios

Our results showed that increasing the reference population size, defined as the number of genotyped individuals with phenotypic records, led to higher genomic prediction accuracy. However, when selective genotyping was applied, the average accuracies were lower compared to scenarios with random genotyping. Without a sufficiently large reference population, the SNP effects may not be well predicted, resulting in poor estimates of breeding values in the validation population and, consequently, lower GEBV prediction accuracy (Goddard and Hayes, 2009). Another factor that explains our linear trends is that the reference population was strongly genetically related to the validation set and was not composed of isolated subpopulations, which made GEBV more reliable (Isidro et al., 2015; Wu et al., 2015). Furthermore, we maintained the same proportional increase based on the genotyping proportion in the size of the validation population as the training population. In other words, the ratio of the animals in the validation and training population with genomic information remained constant as the proportion of animals with genotypes increased. In the case of a validation set of fixed size, we might have a plateau where the increment in accuracy reduces as the number of animals in the training population increases.

Importantly, all genotyping proportions were equally applied for each RC, which provided equal representation of animals of different ages. When this criterion was not included, bias almost doubled, the prediction accuracies reduced on average 0.2, and the dispersion values were even greater towards inflation of GEBV for young animals in all scenarios with selective genotyping. The result of this test reinforced the need to constantly update the reference population with animals in new generations and to have multiple generations represented in the dataset. Only by adopting this strategy will the accuracy of predictions remain constant or increase over time in a breeding program (Pszczola and Calus, 2016). A similar pattern was found in a simulation study of purebred swine with the same heritability values of 0.10 and 0.35 as in the current study (Li et al., 2019). As the proportions of animals increased, the accuracy of prediction increased by applying the same genotyping proportions for each generation. When selective genotyping is preferred, our results indicate that prioritizing genotyping in only one sex—ensuring that at least 40% of animals within that sex are genotyped—can help reduce bias, improve accuracy, and bring dispersion closer to 1. These findings suggest that strategic genotyping of specific sexes or increasing genotyping proportions beyond 40% can enhance the reliability of genetic evaluations in breeding programs.

The increase in bias under the selective genotyping strategy when up to 40% of one sex was genotyped was a consequence of directionally selecting a subset of the population to genotype. Under both an EBV- and phenotypic-based strategy, genotyped animals tend to originate from the same high-ranking families across RC. In our simulation design, selection of replacement animals was strictly based on phenotypic and EBV distribution, consistently favoring higher values, i.e., the highest-ranking animals available. Since we applied directional selection for only one trait, this reinforced the tendency for genotyped animals to originate from the same high-ranking families across cycles. In other words, over successive RC, selection decisions for replacements and culls followed the same criteria as the selective genotyping strategy. As genotyping levels increase incrementally, selection remains concentrated within these families, meaning that while more animals are added, genetic diversity in the reference population does not necessarily increase and can even decrease. This repeated selective genotyping increased bias in genomic prediction. However, as genotyping proportions continue to increase, the inclusion of animals from lower-performing families rises, resulting in a more representative sample and a subsequent reduction in bias.

### 4.5 Genotyping proportion, pedigree, and sex scenarios

All our results showed that genotyping males and females in equal proportion resulted in greater accuracy compared to genotyping animals from a single sex. A similar result was observed in broilers (Lourenco et al., 2015), where it was concluded that both sexes should be genotyped when building reference populations. Our results indicate that prioritizing male genotyping up to 10% is a practical strategy for composite sheep populations, particularly when the cost of genotyping is relatively high. However, this may be trait specific. In a study evaluating genotyping strategies in Latxa dairy sheep, Granado-Tajada et al. (2021) found that genotyping strategies can have a significant impact on prediction accuracy. Specifically, they observed that genotyping only females yielded significantly higher accuracies than genotyping only males, highlighting the importance of considering sex-specific strategies depending on the traits of interest and population structure. In our study, we observed a similar trend, particularly when females were the only sex genotyped. Accuracy was higher when at least 40% of females were genotyped under the random genotyping strategy and when at least 10% were genotyped under the selective strategy based on EBV or phenotype. Our simulation considered that all animals in the reference population had phenotypes. The small differences observed in the genotyped sexes, therefore, only related to the greater selection pressure in males than in females, and the M:F ratio, which impacted the genetic contribution of each sex to subsequent generations.

Other studies have demonstrated the importance of genotyping females. By doing so the accuracy of genomic prediction was improved in Holstein cattle (Uemoto et al., 2017) and genetic gain increased while inbreeding decreased in simulated Danish Jersey and Red Dairy cattle populations (Thomasen et al., 2020). Despite the importance of female genotyping, these studies were conducted on sex-limited traits, and their conclusions are not directly comparable to our study. These findings highlight the need for further research using real data, especially for U.S. sheep breeds where maternal traits, measured only in females, are a priority.

In our study, female genotyping demonstrated additional value by reducing bias and dispersion. The bias tended to reduce as the proportion of genotyped animals increased. However, when only males were genotyped, the bias reduction was less compared to genotyping only females. This primarily happened because males tend to sire more offspring, leading to a more unequal distribution of alleles in the next-generation, reducing the representation of the overall genetic diversity in a population (Falconer and Mackay, 1996). Consequently, genotyping only males primarily captures the genetic variation of a few influential sires, resulting in biased allele frequency estimates and less effective genomic predictions. In addition, females generally represent a broader genetic base because there are more breeding females contributing to the population.

The bias for either pedigree error type tended to stay the same or increase when up to 40% of animals were genotyped. When only females were genotyped, inflation values were closer to the scenario with both males and females, while when only males were genotyped, the inflation was higher. These results emphasize the importance of carefully designing genotyping strategies, particularly considering female genotyping when aiming to reduce bias, improve prediction accuracy, and better capture the overall genetic diversity in genomic evaluations.

## 5 Conclusion

The use of the ssGBLUP method enabled the mitigation of up to 5% of misidentified sires and missing pedigree information, generating higher prediction accuracy than BLUP with a perfect pedigree, while also reducing bias with similar dispersion values. These results indicate that ssGBLUP should be prioritized over traditional BLUP evaluations.

When selective genotyping was used, bias and dispersion increased, and in the long term, breeding programs may experience a reduction in the accuracy of predictions. This reduction can ultimately slow genetic progress if a robust reference population is not established first.

Strategically, the most effective approach to constructing a reference population was random genotyping, with animals selected in a balanced and consistent manner within each reproductive cycle (i.e., production year in real sheep populations). This strategy provided the highest GEBV prediction accuracy and the lowest bias compared to selective approaches.

When genotyping costs are prohibitive, and the trait of interest is measured in both sexes, prioritizing males for genotyping initially is the most efficient approach. However, after reaching a threshold (e.g., 10% of males genotyped), prioritizing females leads to higher prediction accuracy, lower bias, and improved dispersion compared to continued male genotyping.

Overall, these findings highlight the importance of tailoring genotyping strategies based on the proportion of animals genotyped, the traits of interest, and the structure of the target population. They also reinforce the value of ssGBLUP as a robust method for genomic evaluations in sheep breeding programs facing pedigree errors and limited genotyping resources.

## Data Availability

The data and results that support the conclusions of the simulation are fully available within this article and its Supplementary Material. The real-world datasets analyzed in this study are not publicly available because they are the property of U.S. sheep producers and contain commercially sensitive information. Requests to access these datasets may be directed to the corresponding author and will be considered on a case-by-case basis.
