# Supplementary material for: Genotyping strategies for single-step genomic predictions in a simulated sheep population under different scenarios of pedigree error types
Source: Front Genet. 2025 Nov 10;16:1697103. doi: 10.3389/fgene.2025.1697103 (PMC12640758; doi:10.3389/fgene.2025.1697103)
Supplement: Supplementary file 1 [file DataSheet1.docx]

Supplementary Material

**Supplementary Figure S1.** All evaluated genomic prediction scenarios. Blue arrows represent how the scenarios were applied for eight possible outcomes of proportions of genotyped males with 15% of genotyped females, 5% of missing pedigree, 5% of misidentified sires, Genotyping animals with best phenotypes, and with a trait heritability level of 0.10.


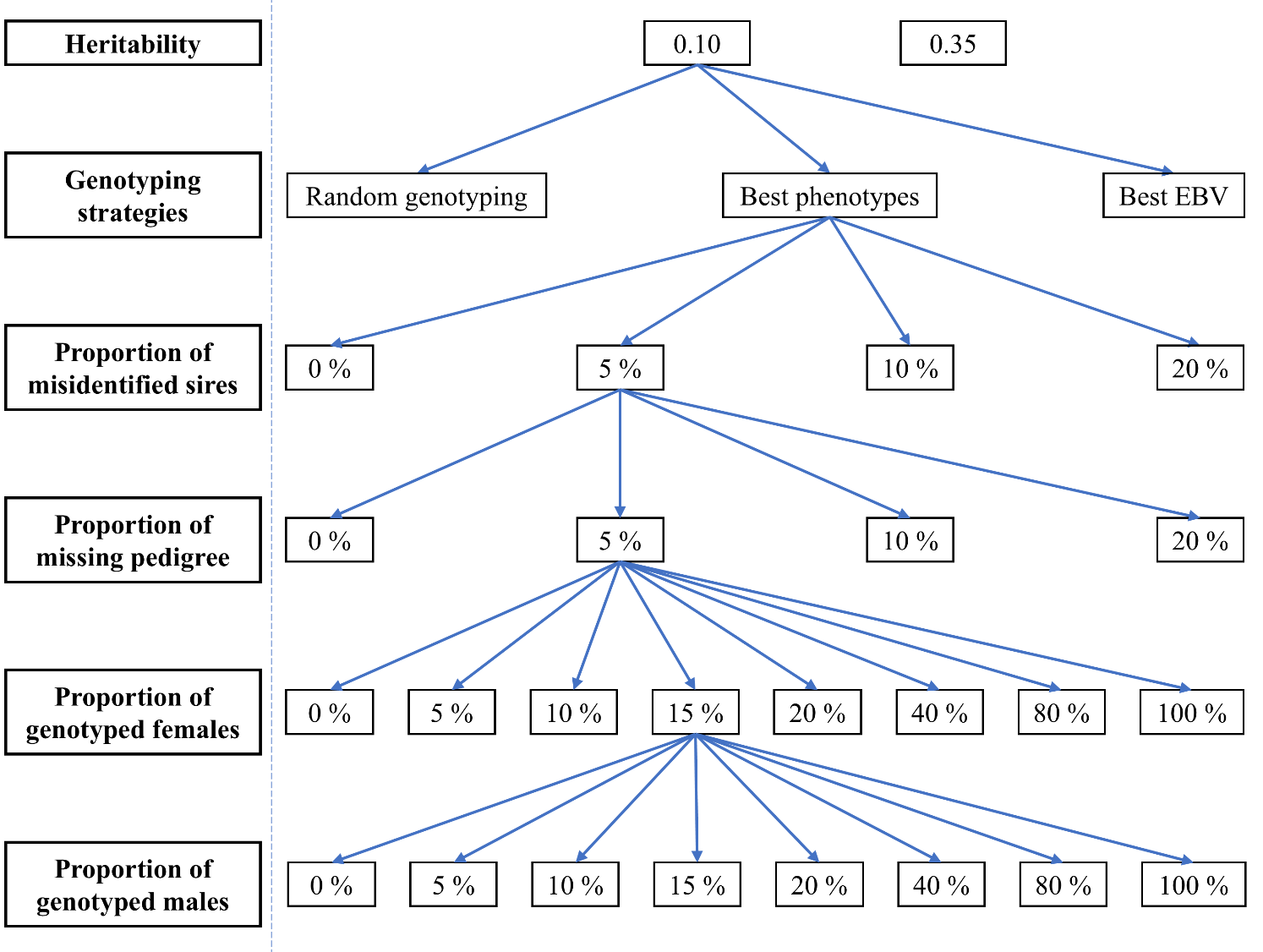


**Supplementary Figure S2.** Average true bias of genomic prediction with their respective percent change for a trait with a heritability level of 0.35 by pedigree error scenarios. Misidentified sire (MS) and missing information (MI) were each evaluated at levels of 0, 0.05, 0.10, and 0.20 and results from their interaction are shown. Panels **(A)** and **(B)** show the true bias results and their percent change compared to the BLUPPP scenario (baseline), respectively. Baseline value is 1.358 **(B)**. Within each panel, pedigree error scenarios with no common superscript are different (*P* < 0.05).


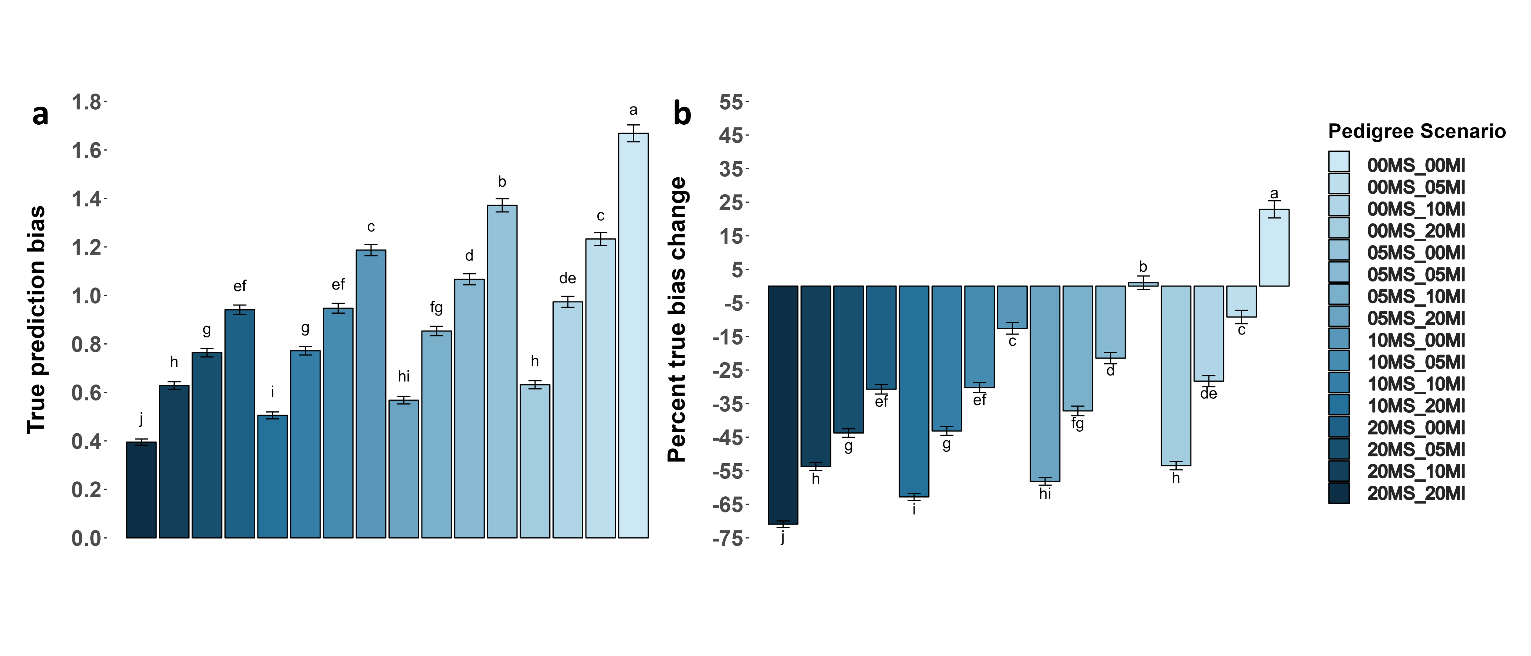


**Supplementary Figure S3.** Average true dispersion of genomic prediction with their respective percent change for a trait with a heritability level of 0.35 by pedigree error scenarios. Misidentified sire (MS) and missing information (MI) were each evaluated at levels of 0, 0.05, 0.10, and 0.20 and results from their interaction are shown. Panels **(A)** and **(B)** show the true dispersion results and their percent change compared to the BLUPPP scenario (baseline), respectively. Baseline value is 0.9408 **(B)**. Within each panel, pedigree error scenarios with no common superscript are different (*P* < 0.05).


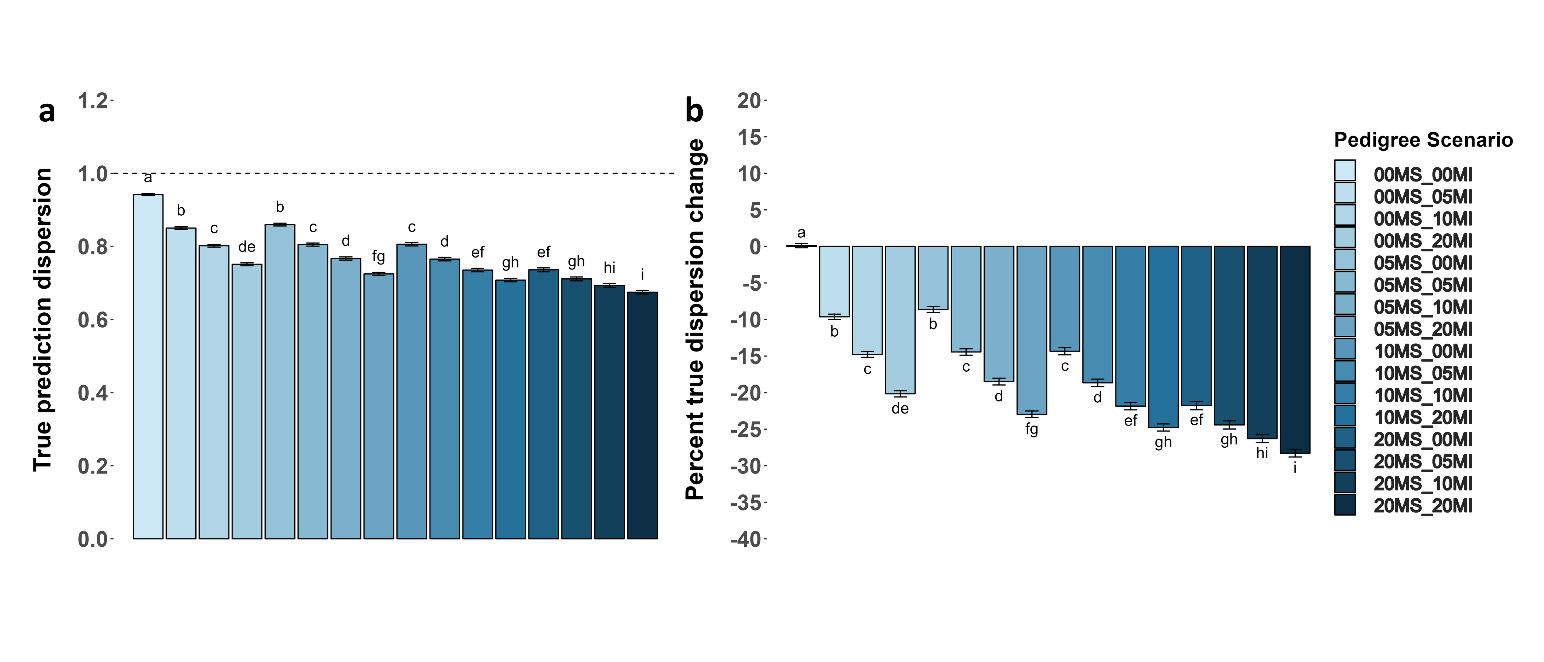


**Supplementary Figure S4.** The average true bias of genomic predictions in simulations for a trait with a heritability level of 0.35 by Genotyping Proportions Scenarios: Interaction between proportions of males genotyped and proportions of females genotyped, all possible combinations of 0, 5, 10, 15, 20, 40, 80, and 100% in both factors. Panel **(A)** shows the true bias results for the EBV genotyping criteria without pedigree errors. Panel **(B)** shows the true bias results for the EBV genotyping criteria with 20% of misidentified sires and 20% of missing information. Panel **(C)** shows the true bias results for the Random genotyping criteria without pedigree errors. Panel **(D)** shows the true bias results for the random genotyping criteria, with 20% misidentified sires and 20% missing information.


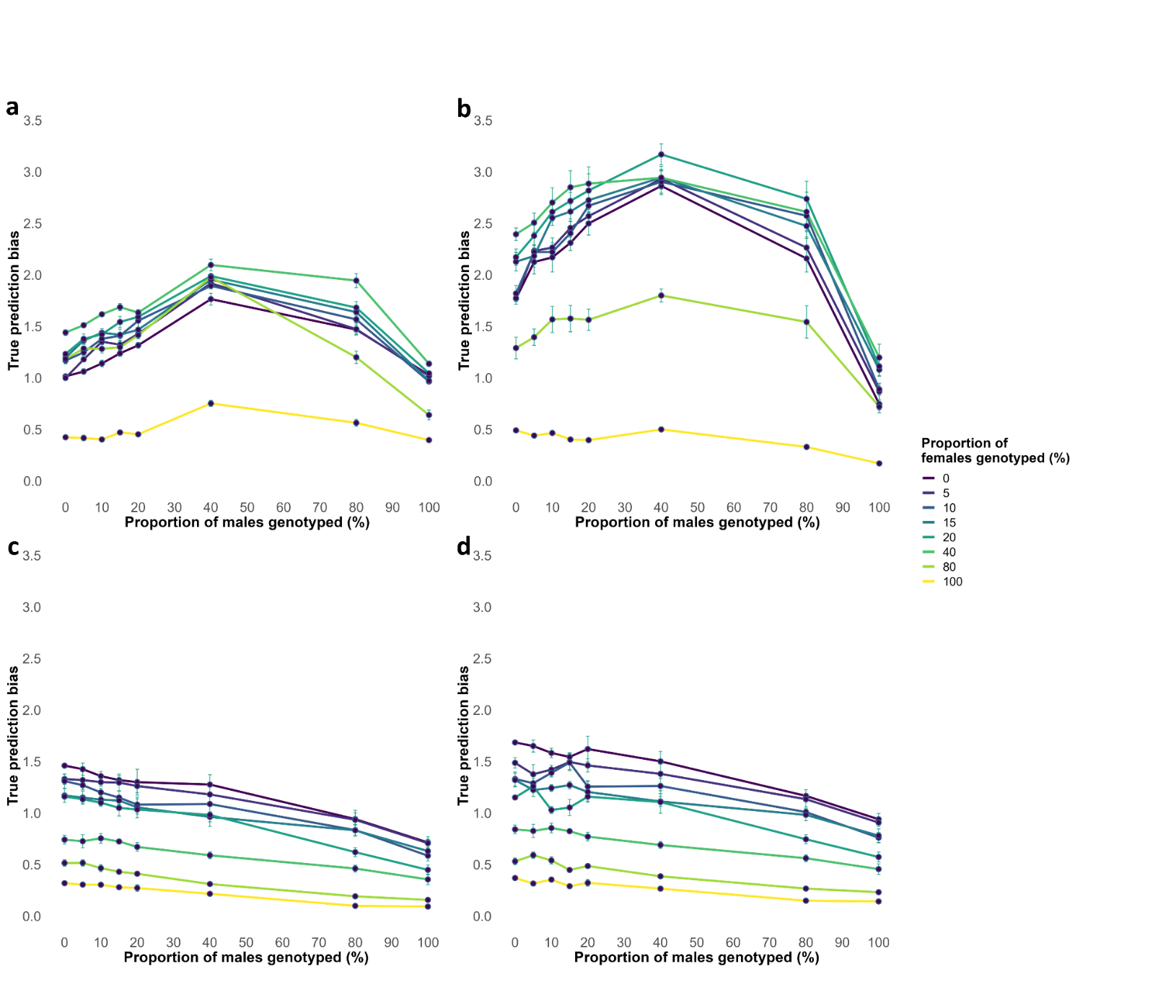


**Supplementary Figure S5.** The average true dispersion of genomic predictions in simulations for a trait with a heritability level of 0.35 by Genotyping Proportions Scenarios: Interaction between proportions of males genotyped and proportions of females genotyped, all possible combinations of 0, 5, 10, 15, 20, 40, 80, and 100% in both factors. Panel **(A)** shows the true dispersion results for the EBV genotyping criteria without pedigree errors. Panel **(B)** shows the true dispersion results for the EBV genotyping criteria with 20% of misidentified sires and 20% of missing information. Panel **(C)** shows the true dispersion results for the Random genotyping criteria without pedigree errors. Panel **(D)** shows the true dispersion results for the random genotyping criteria, with 20% misidentified sires and 20% missing information.


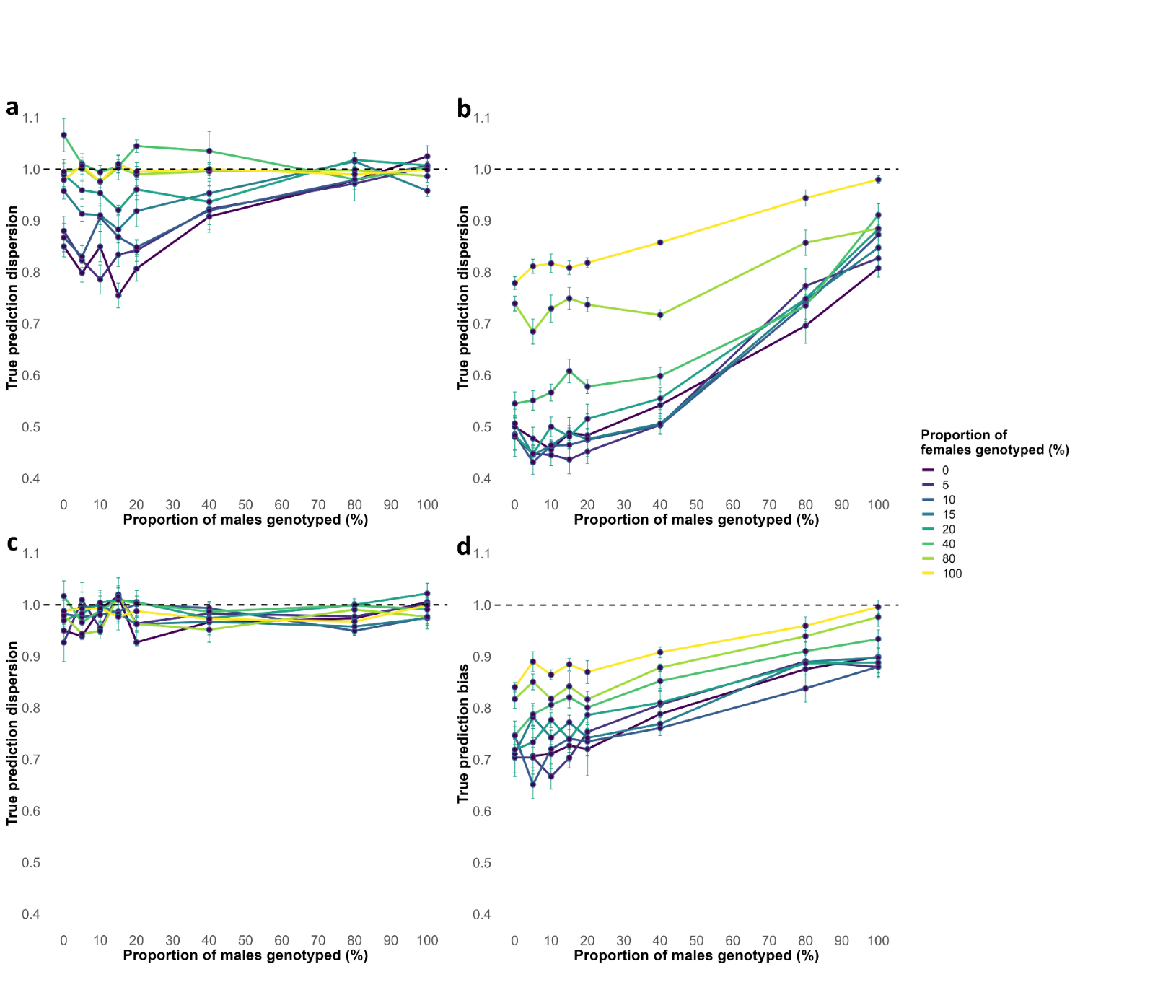


**Supplementary Figure S6.** The average true bias of genomic predictions in simulations for a trait with a heritability level of 0.35 by Genotyping Proportions and Pedigree Scenarios: Interaction between proportions of males (M) and females (F) genotyped, with scenarios without pedigree errors, with 20% of misidentified sires (MS) or with 20% of missing information (MI). Combinations of 5, 10, 20, 40, 80, and 100% of both males and females were genotyped, and the same proportions were applied for each sex separately. Panel **(A)** shows the true bias results for the Random genotyping criteria, and **(B)** shows the true bias results for the EBV genotyping criteria.


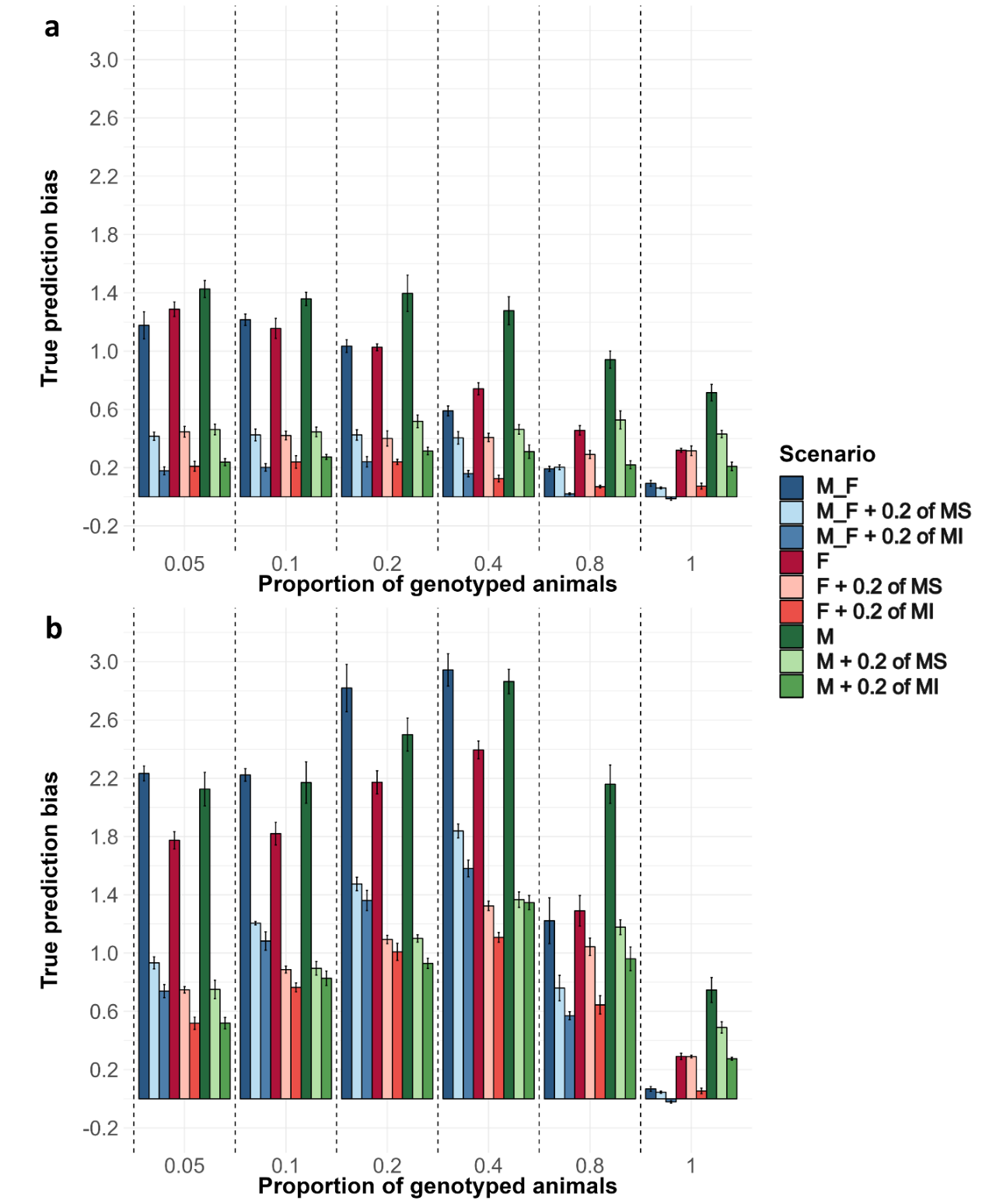


**Supplementary Figure S7.** The average true dispersion of genomic predictions in simulations for a trait with a heritability level of 0.35 by Genotyping Proportions and Pedigree Scenarios: Interaction between proportions of males (M) and females (F) genotyped, with scenarios without pedigree errors, with 20% of misidentified sires (MS) or with 20% of missing information (MI). Combinations of 5, 10, 20, 40, 80, and 100% of both males and females were genotyped, and the same proportions were applied for each sex separately. Panel **(A)** shows the true dispersion results for the Random genotyping criteria, and **(B)** shows the true dispersion results for the EBV genotyping criteria.


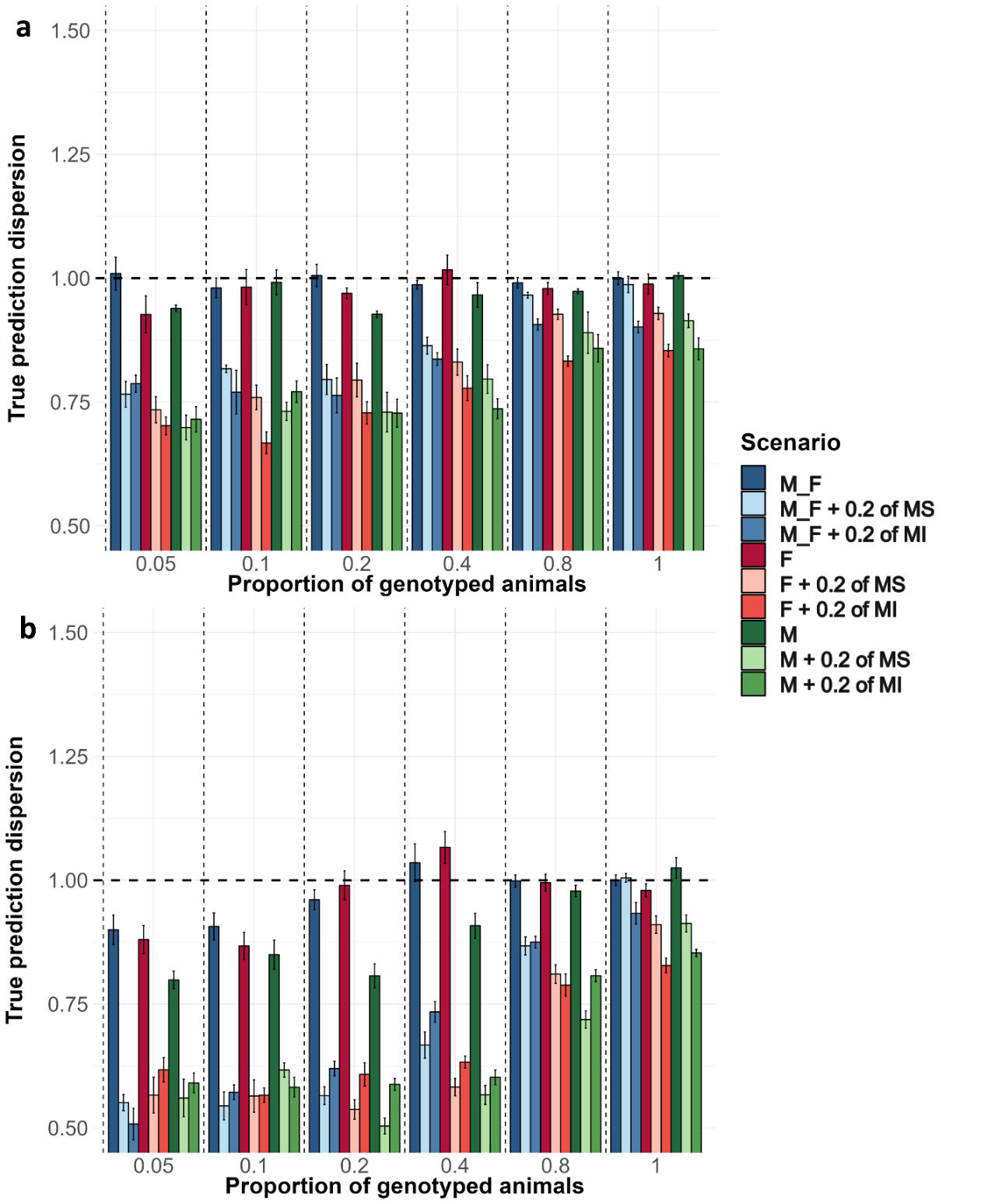


**Supplementary Table S1.** Average (SE) estimated additive genetic variance ($\hat{\sigma}_{a}^{2}$), estimated residual variance ($\hat{\sigma}_{e}^{2}$), estimated heritability ($\hat{h}^{2}$), additive genetic variance ($\sigma_{a}^{2}$), residual variance ($\sigma_{e}^{2}$), and heritability ($h^{2}$) for two traits in each reproductive cycle of a simulated composite sheep population.

| Simulated heritability | General evaluation^a^ | $\hat{\sigma}_{a}^{2}$ | $\hat{\sigma}_{e}^{2}$ | $\hat{h}^{2}$ | $\sigma_{a}^{2}$ | $\sigma_{e}^{2}$ | $h^{2}$ |
| --- | --- | --- | --- | --- | --- | --- | --- |
| 0.35 | First | 1.83 (0.17) | 2.57 (0.10) | 0.41 (0.03) | 1.55 (0.07) | 2.85 (0.01) | 0.35 (0.01) |
|  | Second | 1.64 (0.10) | 2.63 (0.03) | 0.38 (0.02) | 1.48 (0.07) | 2.78 (0.01) | 0.35 (0.01) |
|  | Third | 1.51 (0.08) | 2.71 (0.02) | 0.36 (0.01) | 1.54 (0.09) | 2.76 (0.01) | 0.36 (0.01) |
|  | Fourth | 1.51 (0.08) | 2.71 (0.02) | 0.36 (0.01) | 1.59 (0.11) | 2.76 (0.01) | 0.37 (0.02) |
|  | Fourth (G) | 1.49 (0.09) | 2.78 (0.02) | 0.34 (0.02) | 1.59 (0.11) | 2.76 (0.01) | 0.37 (0.02) |
| 0.10 | First | 1.59 (0.17) | 13.92 (0.19) | 0.10 (0.01) | 1.84 (0.06) | 13.68 (0.15) | 0.12 (<0.00) |
|  | Second | 1.64 (0.11) | 13.49 (0.11) | 0.11 (0.01) | 1.71 (0.04) | 13.41 (0.09) | 0.11 (<0.00) |
|  | Third | 1.68 (0.10) | 13.41 (0.16) | 0.11 (0.01) | 1.74 (0.02) | 13.41 (0.08) | 0.11 (<0.00) |
|  | Fourth | 1.68 (0.10) | 13.41 (0.16) | 0.11 (0.01) | 1.85 (0.03) | 13.54 (0.10) | 0.12 (<0.00) |
|  | Fourth (G) | 1.53 (0.06) | 13.50 (0.12) | 0.10 (<0.00) | 1.85 (0.03) | 13.54 (0.10) | 0.12 (<0.00) |

^a^First, second, and third: general evaluations happening in all scenarios and replicates; Fourth: extra general evaluation for scenarios where the genotyping strategy was best EBV; Fourth (G): general evaluation including genomic information.

**Supplementary Table S2:** Length of individual autosomal chromosome in base pairs (bp), number of segregating sites per chromosome, number of single nucleotide polymorphisms (SNPs) per chromosome, number of quantitative trait loci (QTLs) per chromosome of the simulated genome.

| Chromosome number | Chromosome length (bp) | Number of segregating sites | Number of SNPs | Number of QTLs |
| --- | --- | --- | --- | --- |
| 1 | 275,406,953 | 14,145 | 11,016 | 771 |
| 2 | 248,966,461 | 12,788 | 9959 | 697 |
| 3 | 223,996,068 | 11,505 | 8960 | 627 |
| 4 | 119,216,639 | 6124 | 4769 | 334 |
| 5 | 107,836,144 | 5538 | 4313 | 302 |
| 6 | 116,888,256 | 6004 | 4676 | 327 |
| 7 | 100,009,711 | 5136 | 4000 | 280 |
| 8 | 90,615,088 | 4655 | 3625 | 254 |
| 9 | 94,583,238 | 4858 | 3783 | 265 |
| 10 | 86,377,204 | 4437 | 3455 | 242 |
| 11 | 62,170,480 | 3194 | 2487 | 174 |
| 12 | 79,028,859 | 4059 | 3161 | 221 |
| 13 | 83,079,144 | 4268 | 3323 | 233 |
| 14 | 62,568,341 | 3214 | 2503 | 175 |
| 15 | 80,783,214 | 4149 | 3231 | 226 |
| 16 | 71,693,149 | 3686 | 2868 | 201 |
| 17 | 72,251,135 | 3711 | 2890 | 202 |
| 18 | 68,494,538 | 3519 | 2740 | 192 |
| 19 | 60,445,663 | 3105 | 2418 | 169 |
| 20 | 51,176,841 | 2628 | 2047 | 143 |
| 21 | 49,987,992 | 2568 | 2000 | 140 |
| 22 | 50,780,147 | 2608 | 2031 | 142 |
| 23 | 62,282,865 | 3198 | 2491 | 174 |
| 24 | 42,034,648 | 2159 | 1681 | 118 |
| 25 | 45,223,504 | 2324 | 1809 | 127 |
| 26 | 44,047,080 | 2262 | 1762 | 123 |

**Supplementary Table S3:** Average number of genotyped males (M) and females (F) in training and validation sets across genotyping proportion scenarios.

| Proportion of genotyped males | Proportion of genotyped females | Average number of genotyped animals | | | | |
| --- | --- | --- | --- | --- | --- | --- |
|  |  | Training set^a^ | | | Validation set | |
|  |  | M | F | M | | F |
| 0 | 0 | 0 | 0 | 0 | | 0 |
| 5 | 0 | 284 | 0 | 129 | | 0 |
| 10 | 0 | 568 | 0 | 257 | | 0 |
| 15 | 0 | 852 | 0 | 385 | | 0 |
| 20 | 0 | 1136 | 0 | 513 | | 0 |
| 40 | 0 | 2271 | 0 | 1025 | | 0 |
| 80 | 0 | 4541 | 0 | 2050 | | 0 |
| 100 | 0 | 5676 | 0 | 2562 | | 0 |
| 0 | 5 | 0 | 398 | 0 | | 129 |
| 5 | 5 | 284 | 398 | 129 | | 129 |
| 10 | 5 | 568 | 398 | 257 | | 129 |
| 15 | 5 | 852 | 398 | 385 | | 129 |
| 20 | 5 | 1136 | 398 | 513 | | 129 |
| 40 | 5 | 2271 | 398 | 1025 | | 129 |
| 80 | 5 | 4541 | 398 | 2050 | | 129 |
| 100 | 5 | 5676 | 398 | 2562 | | 129 |
| 0 | 10 | 0 | 795 | 0 | | 257 |
| 5 | 10 | 284 | 795 | 129 | | 257 |
| 10 | 10 | 568 | 795 | 257 | | 257 |
| 15 | 10 | 852 | 795 | 385 | | 257 |
| 20 | 10 | 1136 | 795 | 513 | | 257 |
| 40 | 10 | 2271 | 795 | 1025 | | 257 |
| 80 | 10 | 4541 | 795 | 2050 | | 257 |
| 100 | 10 | 5676 | 795 | 2562 | | 257 |
| 0 | 15 | 0 | 1193 | 0 | | 385 |
| 5 | 15 | 284 | 1193 | 129 | | 385 |
| 10 | 15 | 568 | 1193 | 257 | | 385 |
| 15 | 15 | 852 | 1193 | 385 | | 385 |
| 20 | 15 | 1136 | 1193 | 513 | | 385 |
| 40 | 15 | 2271 | 1193 | 1025 | | 385 |
| 80 | 15 | 4541 | 1193 | 2050 | | 385 |
| 100 | 15 | 5676 | 1193 | 2562 | | 385 |
| 0 | 20 | 0 | 1590 | 0 | | 513 |
| 5 | 20 | 284 | 1590 | 129 | | 513 |
| 10 | 20 | 568 | 1590 | 257 | | 513 |
| 15 | 20 | 852 | 1590 | 385 | | 513 |
| 20 | 20 | 1136 | 1590 | 513 | | 513 |
| 40 | 20 | 2271 | 1590 | 1025 | | 513 |
| 80 | 20 | 4541 | 1590 | 2050 | | 513 |
| 100 | 20 | 5676 | 1590 | 2562 | | 513 |
| 0 | 40 | 0 | 3179 | 0 | | 1025 |
| 5 | 40 | 284 | 3179 | 129 | | 1025 |
| 10 | 40 | 568 | 3179 | 257 | | 1025 |
| 15 | 40 | 852 | 3179 | 385 | | 1025 |
| 20 | 40 | 1136 | 3179 | 513 | | 1025 |
| 40 | 40 | 2271 | 3179 | 1025 | | 1025 |
| 80 | 40 | 4541 | 3179 | 2050 | | 1025 |
| 100 | 40 | 5676 | 3179 | 2562 | | 1025 |
| 0 | 80 | 0 | 6358 | 0 | | 2050 |
| 5 | 80 | 284 | 6358 | 129 | | 2050 |
| 10 | 80 | 568 | 6358 | 257 | | 2050 |
| 15 | 80 | 852 | 6358 | 385 | | 2050 |
| 20 | 80 | 1136 | 6358 | 513 | | 2050 |
| 40 | 80 | 2271 | 6358 | 1025 | | 2050 |
| 80 | 80 | 4541 | 6358 | 2050 | | 2050 |
| 100 | 80 | 5676 | 6358 | 2562 | | 2050 |
| 0 | 100 | 0 | 7947 | 0 | | 2562 |
| 5 | 100 | 284 | 7947 | 129 | | 2562 |
| 10 | 100 | 568 | 7947 | 257 | | 2562 |
| 15 | 100 | 852 | 7947 | 385 | | 2562 |
| 20 | 100 | 1136 | 7947 | 513 | | 2562 |
| 40 | 100 | 2271 | 7947 | 1025 | | 2562 |
| 80 | 100 | 4541 | 7947 | 2050 | | 2562 |
| 100 | 100 | 5676 | 7947 | 2562 | | 2562 |

^a^The reported average number of genotyped animals is equally divided into the three reproductive cycles of the training set.

**Supplementary Table S4:** Overview of the parameters used for scenario comparison^a^**.**

| Parameter^b^ | Formula^c^ |
| --- | --- |
| $acc$ | $\frac{cov\left( u,\hat{u} \right)}{\sqrt{var\left( u \right)var\left( \hat{u} \right)\text{ }}}$ |
| $bias$ | $\bar{u}-\bar{\hat{u}}$ |
| $disp$ | $cov(u,\hat{u})/var(\hat{u}$) |
| $acc percentage change$ | $\frac{{acc}_{ssGBLUP}-{acc}_{BLUPPP}}{{acc}_{BLUPPP}}$ |
| $bias percentage change$ | $\frac{{bias}_{ssGBLUP}-{bias}_{BLUPPP}}{{bias}_{BLUPPP}}$ |
| $disp percentage change$ | $\frac{{disp}_{ssGBLUP}-{disp}_{BLUPPP}}{{disp}_{BLUPPP}}$ |

^a^All true statistics calculated with the true breeding value (TBV) and estimated breeding value (EBV) or genomic estimated breeding value (GEBV). Percentage changes calculated comparing the average statistics of the evaluated single-step Genomic Best Linear Unbiased Prediction (ssGBLUP) scenarios against the Best Linear Unbiased Prediction (BLUP) with a perfect pedigree (BLUPPP).

^b^Parameter: acc: prediction accuracy; bias: prediction bias; disp: prediction dispersion.

^c^Formula: $cov\left( u,\hat{u} \right)$ is the covariance between TBV ($u$) and GEBV (or EBV) ($\hat{u}$), $var\left( u \right)$and $var\left( \hat{u} \right)$ are the variance of TBV and GEBV (or EBV), respectively, $\bar{u}$ and $\bar{\hat{u}}$ are the averages of TBV and GEBV (or EBV), respectively, all from the validation sets. ${acc}_{ssGBLUP}$ is the average acc value for the evaluated ssGBLUP scenario, ${acc}_{BLUPPP}$ is the acc value for BLUPPP respective scenario. ${bias}_{ssGBLUP}$ is the average bias value for the evaluated ssGBLUP scenario, ${bias}_{BLUPPP}$ is the bias value for BLUPPP respective scenario. ${disp}_{ssGBLUP}$ is the average disp value for the evaluated ssGBLUP scenario, ${disp}_{BLUPPP}$ is the disp value for BLUPPP respective scenario.
